# Image-based compound profiling reveals a dual inhibitor of tyrosine kinase and microtubule polymerization

**DOI:** 10.1038/srep25095

**Published:** 2016-04-27

**Authors:** Kenji Tanabe

**Affiliations:** 1 Medical Research Institute, Tokyo Women’s Medical University, Tokyo 162-8666, Japan

## Abstract

Small-molecule compounds are widely used as biological research tools and therapeutic drugs. Therefore, uncovering novel targets of these compounds should provide insights that are valuable in both basic and clinical studies. I developed a method for image-based compound profiling by quantitating the effects of compounds on signal transduction and vesicle trafficking of epidermal growth factor receptor (EGFR). Using six signal transduction molecules and two markers of vesicle trafficking, 570 image features were obtained and subjected to multivariate analysis. Fourteen compounds that affected EGFR or its pathways were classified into four clusters, based on their phenotypic features. Surprisingly, one EGFR inhibitor (CAS 879127-07-8) was classified into the same cluster as nocodazole, a microtubule depolymerizer. In fact, this compound directly depolymerized microtubules. These results indicate that CAS 879127-07-8 could be used as a chemical probe to investigate both the EGFR pathway and microtubule dynamics. The image-based multivariate analysis developed herein has potential as a powerful tool for discovering unexpected drug properties.

Many small-molecule compounds are used as inhibitors of cellular signaling pathways and therapeutic agents^1^,^2^,^3^. In both basic research and clinical settings, elucidating the target selectivity of such compounds is critical for predicting and interpreting their effects^4^,^5^,^6^,^7^. A library of kinases, for example, might be useful for measuring the effects of compounds on kinase activities and identifying the target kinase of each compound^4^,^8^,^9^,^10^. Through such approaches, it has become clear that most compounds, including many drugs in clinical use, have multiple targets. Protein libraries make it possible to screen many proteins simultaneously, but the number of proteins available in such systems is still limited relative to the diversity of proteins within living cells. Consequently, it is possible that a given compound of interest may have an unexpected target inside cells. If an as yet unknown protein is revealed as a new target, such information could explain a compound’s side effects or encourage repositioning of the compound as a treatment for other diseases^11^,^12^.

In this study, I focused on epidermal growth factor receptor (EGFR), a prototypical receptor tyrosine kinase (RTK), because this protein has been extensively investigated as an important target of small-molecule compounds in both basic and clinical research^13^,^14^,^15^. Inhibitors of EGFR tyrosine kinase used in clinical practice include gefitinib, erlotinib, and afatinib, which are used in therapy against non-small cell lung cancers (NSCLCs) harboring EGFR mutations^16^,^17^,^18^. In addition to direct inhibitors of EGFR itself, compounds that affect EGFR signaling components such as K-Ras, MEK1, and PI3KCA are also candidate therapeutic tools for use against NSCLCs^19^,^20^,^21^. Furthermore, because the subcellular localization of RTKs regulate the downstream fate of RTK-elicited signals, the intracellular machineries involved in vesicle transport also represent potential targets of anti-cancer drugs^15^,^22^,^23^.

Several previous trials inferred a novel/hidden target of small-molecule compounds^7^,^24^,^25^,^26^. In this study, I developed a quantitative, and statistical method to analyze microscopically obtained EGFR-related images. Fourteen inhibitors associated with signal transduction and intracellular trafficking of EGFR can be hierarchically classified based on their effects on cellular phenotype. I discovered that a 4,6-dianilinopyrimidine EGFR inhibitor (CAS 879127-07-8), the most uni-specific inhibitor among the various currently available kinase inhibitors^27^,^28^, was co-classified in the same cluster as the microtubule depolymerizer nocodazole. In fact, this compound induced microtubule depolymerization in both biochemical and cell-based assays. These data indicate that CAS 879127-07-8 could be used as a chemical probe to investigate the EGFR pathway and microtubule dynamics. The image-based multivariate analysis developed herein has potential as a powerful tool for discovering unanticipated drug properties.

## Results

### Quantitative analysis of signal transduction and intracellular traffic of EGF/EGFR

**Figure 1:**
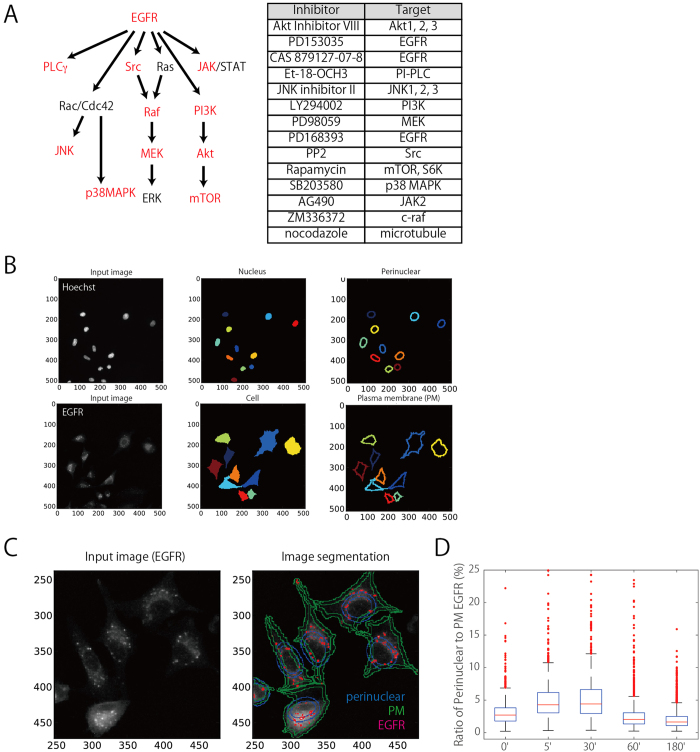
Compounds and quantitative analyses. (**A**) Inhibitors, components of the EGFR inhibitor panel (Merck Millipore), and nocodazole were used in this study. Primary targets of EGFR inhibitor panel are indicated in red. (**B**) Image segmentation and identification of objects to establish regions of interest (ROIs). DNA staining and GFP-EGFR signals were used to define the ‘nucleus’ and ‘cell’ regions, respectively. The ‘nucleus’ and ‘cell’ regions were expanded and shrunk, respectively, and the resultant areas (after subtraction of the original objects) were defined as ‘perinuclear’ and ‘PM’. (**C**) An example of image segmentation. The input image of EGFR was segmented into the perinuclear (blue) and PM (green) regions, and the signal intensity of GFP-EGFR (magenta) was measured in each region. (**D**) The ratios of EGFR signals in the perinuclear region to those in the PM region, measured in single cells treated with DMSO, were plotted for each incubation time.

To examine the effects of various compounds on cellular phenotypes, I constructed an image-based assay system in which the intensity and intracellular localization of fluorescent signals were measured quantitatively. A549-GFP-EGFR cells, in which the genomic EGFR has been endogenously tagged with GFP, was used in this study. Cells were seeded in 96-well plates and treated for 1 h with inhibitors of EGFR signaling (Fig. 1A). EGF was then added to the culture at 100 ng/ml, a concentration at which EGFR was primarily transported to a degradation pathway^29^,^30^. After incubation for 0, 5, 30, 60, or 180 min, cells were fixed and processed for immunofluorescence using antibodies against molecules implicated in EGFR signaling, including phosphorylated ERK (pERK), phosphorylated Akt (pAkt), and several phosphoinositides (PtdIns(3)P, PtdIns(4)P, and PtdIns(4,5)P2)^22^,^31^,^32^,^33^. In addition, endocytic trafficking was visualized using either EGF or transferrin. EGF was used as a marker to monitor the degradation pathway, whereas transferrin was used to measure the recycling pathway^34^,^35^,^36^. To visualize nuclear DNA, the cells were stained with Hoechst. Images were acquired by automated microscopy. Thus, cell phenotypes were monitored simultaneously using four different markers: GFP-EGFR, two signaling/trafficking molecules, and Hoechst.

Several intracellular regions of interest were defined as illustrated in Fig. 1B. The ‘nucleus’ and ‘cell’ regions represent the area of Hoechst staining and GFP-EGFR fluorescence, respectively. The ‘perinuclear region’ was obtained by expanding the contour of the ‘nucleus’ with a diameter of 7 pixels, whereas the ‘plasma membrane’ region (PM) was obtained by shrinking the contour of the ‘cell’ region with a 5-pixel diameter. These intracellular regions were used to define the intracellular localization of observed signals/proteins^37^. In Fig. 1C, for example, the localization of EGFR was assigned to the ‘perinuclear’ and ‘PM’ regions. After the addition of EGF, I quantitatively monitored the trafficking of GFP-EGFR (Fig. 1D)^35^,^38^. The ratios of EGFR signal intensities from the ‘perinuclear’ region to those from the ‘PM’ region were measured in 500–1000 cells and plotted. As can be seen in the bar plot, EGFR moved toward the perinuclear region after incubation for 5 or 30 min (reflected as an increase in the number of cells with higher perinuclear signals). The regression coefficient (the slope of intensity in ‘perinuclear’ vs. ‘PM’) reached a maximum at 30 min, suggesting that EGFR reached to late endosomes/lysosomes, which localizes on perinuclear region (see Supplementary Fig. S1 as well). After incubation for 60 or 180 min, the number of cells with high signals decreased, indicating that EGFR was degraded. Thus, my selection of intracellular regions was appropriate for measuring the intracellular trafficking of EGFR.

### Comparison of compounds’ effects using the Kolmogorov–Smirnov (KS) test

**Figure 2:**
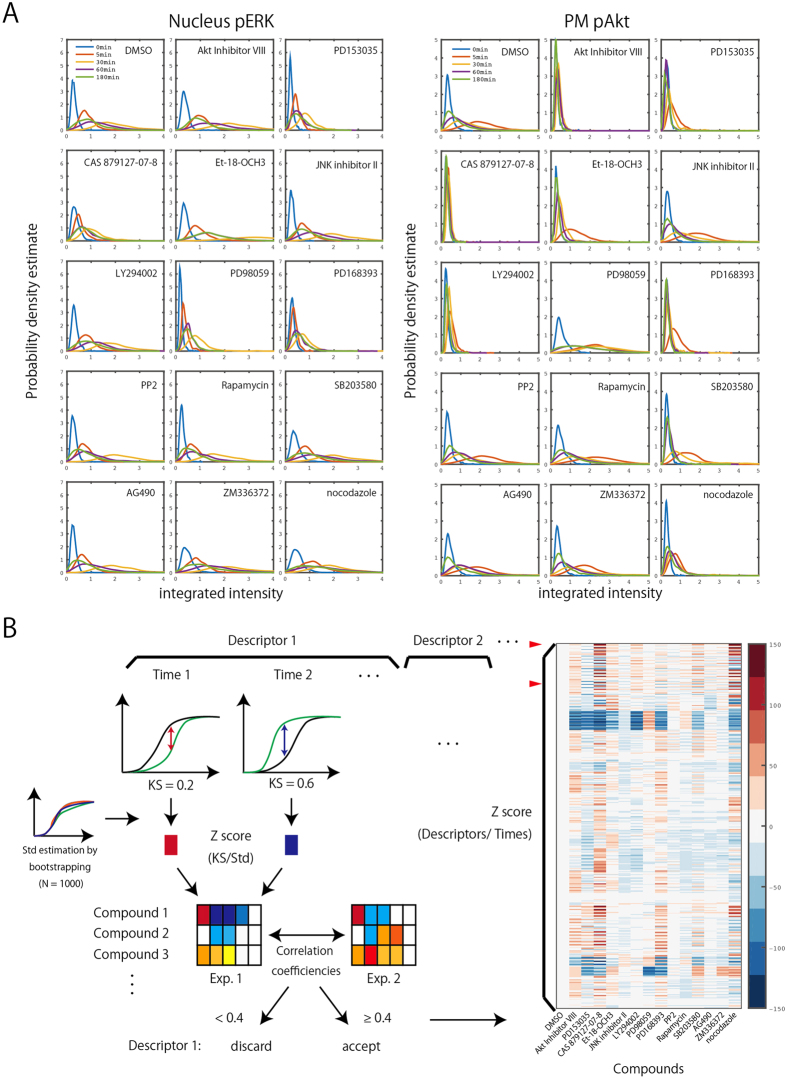
Statistical analysis of the effects of compounds based on their phenotypes. (**A**) Integrated intensity of pERK from the ‘nucleus’ region (Nucleus_pERK) and pAkt from the ‘PM’ (PM_pAkt) were measured in each cell, and kernel estimation was used to calculate the probability density estimation. (**B**) Key steps of algorithm for analyzing compound effects. A one-sided two-sample Kolmogorov–Smirnov (KS) test was performed to compare control and compound-treated cells, and a standardized Z-score was calculated (see experimental methods). Descriptors with Z-scores exhibiting significant correlation between two independent experiments (R > 0.4) were used for subsequent analysis.

Using the system described above, I measured 151 image features (descriptors) listed in Supplementary Table S1. These features were selected comprehensively from the viewpoints of signal transduction and intracellular traffic. Figure 2A shows two examples of such descriptors (Nucleus_pERK and PM_pAkt). EGF stimulation induced phosphorylation of ERK and Akt, and their translocation to the nucleus and PM, respectively. In control cells (DMSO), the signals from Nucleus_pERK and PM_pAkt reached their maxima after stimulation for 30 and 5 min, respectively. Treatment with certain compounds dramatically affected the appearance of these signals. For instance, inhibitors of EGFR (PD153035, CAS 879127-07-8, and PD168393) decreased both Nucleus_pERK and PM_pAkt. On the other hand, Akt Inhibitor VIII (an inhibitor of phosphorylation/activation of Akt) inhibited PM_pAkt but increased Nucleus_pERK, whereas PD98059 (an inhibitor of MEK, which phosphorylates ERK) inhibited Nucleus_pERK and increased PM_pAkt. These observations are consistent with the reported crosstalk between the Akt and ERK pathways^39^. Thus, quantitative image analysis employing descriptors is useful for revealing the effects of inhibitors.

To estimate the effects of inhibitors in a quantitative manner, I adopted the one-sided two-sample KS test (Fig. 2B, left panel)^40^,^41^. Because the conventional KS statistic lacks a sign, a variant statistic reported by Perlman *et al.* was used in this study^24^. A standardized Z-score was calculated by dividing the signed KS value with the standard deviation of the corresponding control KS value, which was calculated by bootstrapping simulation (n = 1000). This standardized/normalized Z-score was used to compare the extent of a compound’s effects on each descriptor.

The reliability of each descriptor was evaluated by calculating Pearson’s correlation coefficient between two independent experiments. If the correlation coefficient was above 0.4, descriptors were used for subsequent analysis; descriptors with lower coefficients were discarded. The correlation coefficients obtained for each descriptor are shown in Supplementary Table S1, and the selected descriptors are listed in Supplementary Table S2. Ultimately, 570 Z-scores were indexed by each of 114 descriptors at each of five time points (Z_d, t_). The Z-score was further indexed for each of 14 compounds (total, 7980 Z_d, c, t_) (Fig. 2B, right panel).

### Cluster analysis of quantitative phenotypes categorized 14 compounds into 4 groups and CAS 879127-07-8 and nocodazole into the same cluster

Although the descriptors adopted in Supplementary Table S2 appear to represent many different types of information, it is possible that the data obtained for each descriptor may contain biological relevant content. Supplementary Fig. S2 depicts the correlation matrix detected among the Z-scores of 570 descriptors where the existence of positive or negative correlations can be recognized to various degrees. Hierarchical clustering of these correlations then shows seven major or fourteen minor groups (indicated by red and blue dotted lines, respectively, in Supplementary Fig. S3). The existence of such groups roughly suggests the extent of the data complexity. Therefore, I next tried to extract principal components (PCs) that would explain phenotype variability based on combinations of descriptors. Totally, fourteen PCs that could explain 100% of the variance were extracted, and more than 95% of the variance could be explained by seven out of fourteen PCs (Supplementary Fig. S4). Considering both the number of observed molecules and their biological relevance, the detected numbers of groups such as seven or fourteen seems reasonable.

**Figure 3:**
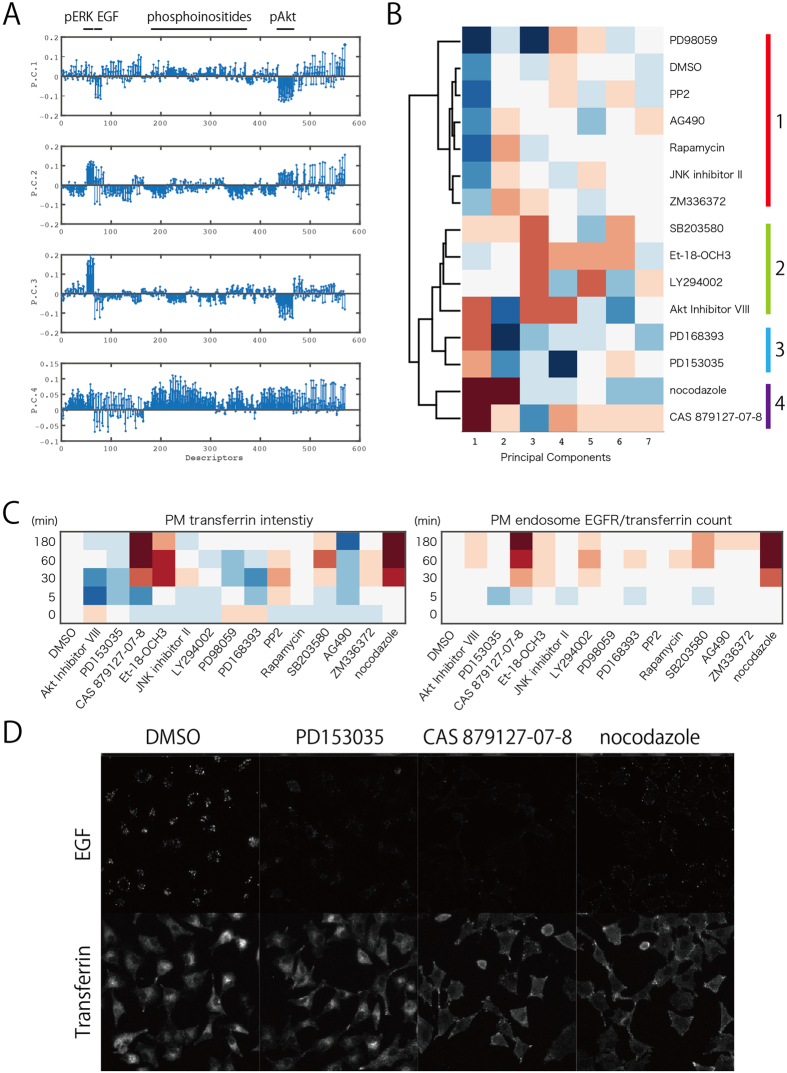
(**A**) Contribution of descriptors to four major principal components. Descriptors are listed in Supplementary Table S1. (**B**) Hierarchical clustering of compounds based on seven principal components. Four main clusters were identified, as indicated by colors on the right side. (**C**) Heat map, based on data in Fig. 2A, shows the effects of inhibitors on PM transferrin intensity (left panel) and the PM endosome EGFR/transferrin count (right panel). (**D**) Cells were treated with the indicated compounds and allowed to internalize Alexa Fluor 647-EGF or Alexa Fluor 647-Tfn for 30 min.

Figure 3A illustrates the relative contribution (score) of each descriptor (total, 570 descriptors) to each PC (four out of seven PCs are shown as representatives). PC1 was characteristic of highly negative scores for pAkt and EGF endocytosis, whereas positive scores for pERK made a large contribution to PC2. Furthermore, PC3 contained positive scores for pERK and negative scores for pAkt, suggesting that this component reflects, at least to some extent, the crosstalk between the ERK and Akt pathways^39^. PC4 contained highly positive scores for phosphoinositides, including PtdIns(3)P, PtdIns(4)P, and PtdIns(4,5)P2 with some contribution from pAkt, suggesting that this component reflects phosphoinositide-linked pAkt activation^42^.

Using these seven PCs, I hierarchically clustered the observed effects of 14 inhibitors (and DMSO control) by the Euclid distance and Ward methods. As seen in Fig. 3B, four main clusters could be identified (Essentially similar results were obtained when using all of the fourteen PCs. See Supplementary Fig. S5). Because these four clusters were identified based solely on cellular phenotypes, it is possible that compounds falling into the same cluster might share common targets in signal transduction pathways.

One major characteristic of cluster 1 (and one not found in the other clusters) was a low PC1 score. In other words, in cluster 1 (but not in the other clusters), pAkt and EGF endocytosis was highly active. Therefore, compounds in cluster 1, including the DMSO control, are considered to exert relatively limited effects on signal transduction and trafficking of EGF/EGFR. These compounds include PP2 (Src inhibitor), AG490 (JAK inhibitor), and rapamycin (mTOR and S6K inhibitor), JNK inhibitor II, ZM336372, and PD98059. It should be noted that PD98059, an inhibitor of MEK, had an extremely low PC1 score; this compound might have stimulated massive phosphorylation Akt via cross-inhibition^39^.

On the other hand, compounds with high PC1 scores were divided among three clusters. Cluster 2 includes SB203580 (p38 MAPK inhibitor), Et-18-OCH3 (PI-PLC inhibitor), LY294002 (PI3K inhibitor), and Akt Inhibitor VIII. Several compounds affecting pathways upstream of Akt^43^,^44^,^45^,^46^,^47^ are included in this cluster. Cluster 3 contains PD153035 and PD168393; both are inhibitors of EGFR itself^48^,^49^. However, a third inhibitor of EGFR, CAS 879127-07-8^27^, fell into cluster 4 along with nocodazole, an inhibitor of microtubule polymerization^50^, which is reflected to some extent by the high correlation coefficient of the Z-scores between these two compounds (See Supplementary Fig. S6). Because the presence of cluster 4 was totally unexpected, I sought to determine why CAS 879127-07-8 and nocodazole were in the same cluster.

### Nocodazole does not likely target kinases involved in the EGFR pathway

Although CAS 879127-07-8 is a highly uni-specific inhibitor^28^, many other kinase inhibitors have one or more secondary targets in addition to their primary target (for an illustration, see CAS 879127-07-8, gefitinib, and erlotinib in Supplementary Fig. S7A, which depicts a drug profiling panel using recombinant kinases^4^). Thus, I explored the possibility that nocodazole might target a kinase as well as microtubules. The target molecules reported to date for the compounds in clusters 3 and 4 are provided in Supplementary Fig. S7B (data from ChEMBL). Three inhibitors of EGFR share two targets, EGFR and ErbB4, whereas nocodazole has no kinase target. Target prediction using SwissTargetPrediction also indicated that nocodazole does not share any kinase target with CAS 879127-07-8 (Supplementary Fig. S7C). Although one recent study reported that nocodazole is a high-affinity ligand for Abl, c-kit, B-Raf, and MEK^51^, I observed hyperphosphorylation of ERK in nocodazole-treated cells as described by Hayne *et al.*, suggesting that the Raf-MEK-ERK pathway is not likely to be impaired by nocodazole. Thus, it seemed unlikely that CAS 879127-07-8 and nocodazole target a common kinase/pathway.

### CAS 879127-07-8 and nocodazole induce similar cellular phenotypes

PC analyses were adequate to classify inhibitors, as shown in Fig. 3B. However, because each PC was actually a complicated mixture of descriptors, such analysis was not sufficient to characterize each inhibitor in detail. Therefore, I searched one by one for descriptors shared by nocodazole and CAS 879127-07-8 (but not by the other inhibitors of EGFR, PD153035 and PD168393). Two descriptors satisfied these requirements: the intensity of transferrin signals around PM (PM transferrin intensity) and the count of PM endosomes containing EGFR/transferrin (PM endosome EGFR/transferrin count).

As seen in Fig. 3C, both CAS 879127-07-8 and nocodazole increased PM transferrin intensity and the PM endosome EGFR/Tfn count (Fig. 3C and red arrowheads in Fig. 2B, right panel). After internalization from the PM, transferrin is delivered to early endosomes and subsequently to recycling endosomes. Therefore, the observed increase in PM transferrin intensity indicates that internalization of transferrin from the PM was impaired. On the other hand, EGF/EGFR internalized into early endosomes is directed toward a degradation pathway. The observed increase in the PM endosome EGFR/transferrin count means that the sorting of EGFR and transferrin at early endosomes was impaired^35^,^36^, and that these two molecules coexisted in that compartment for a prolonged period of time.

Figure 3D shows a microscopic image of transferrin/EGF behavior. Because transferrin internalization occurs independently of EGFR phosphorylation, PD153035 (and PD168393, not shown) did not affect transferrin internalization (Fig. 3D, bottom panel). By contrast, in cells treated with CAS 879127-07-8 or nocodazole, transferrin accumulated at the PM even after incubation for 30 min. On the other hand, all three compounds (PD153035, CAS 879127-07-8, and nocodazole) impaired EGF internalization (Fig. 3D, upper panels). Both PD153035 and CAS 879127-07-8 inhibit EGFR auto-phosphorylation, a step required for its internalization^52^. By contrast, nocodazole disrupts microtubule organization required for the intracellular transport of internalized vesicles, causing EGF/transferrin to accumulate in the PM/endosome region. Thus, the inhibition of transferrin internalization observed upon treatment with CAS 879127-07-8 might be due not to this compound’s inhibitory effect on EGFR, but instead to a mechanism similar to that of nocodazole.

### CAS 879127-07-8 is a novel microtubule depolymerizer

**Figure 4:**
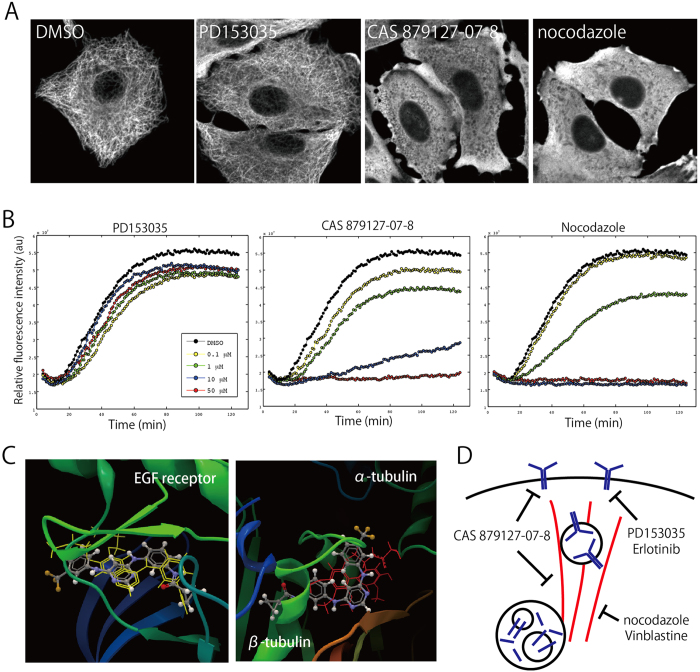
CAS 879127-07-8 is a novel microtubule depolymerizer. (**A**) Cells were treated with indicated compounds for 1 h, fixed, and processed for immunofluorescence using anti-tubulin antibody. Polymerized microtubules appeared in DMSO- and PD153035-tretaed cells, but not in CAS 879127-07-8- or nocodazole-treated cells. (**B**) *In vitro* microtubule polymerization assay confirmed that both CAS 879127-07-8 and nocodazole, but not PD153035, prevented microtubule polymerization in a concentration-dependent manner (center panel). (**C**) Cells were counted after treatment with the indicated compounds for 24 h. Note that CAS 879127-07-8 and nocodazole, but not PD153035, decreased cell number, indicating that these compounds interfered with mitosis.

Based on the above reasoning, I analyzed the possible effects of CAS 879127-07-8 on microtubule organization (Fig. 4A). In control and PD153035-treated cells, I observed a radial array of microtubules. On the other hand, microtubule organization was disrupted in CAS 879127-07-8- and nocodazole-treated cells. Similar results were obtained in seven other cell lines of different genetic backgrounds (Supplementary Fig. S8). One of these cell lines, NCI-H661 does not express endogenous EGFR^53^. Therefore, the observed disruption of microtubules by CAS 879127-07-8 appears to be unrelated to its inhibitory effect on EGFR tyrosine kinase activity. In addition, the addition of CAS 879127-07-8 or nocodazole, but not PD153035, to the culture reduced the cell number, and increased the percentage of cells in G2/M phase, probably because mitosis was impaired (Supplementary Fig. S9). Thus, like nocodazole, CAS 879127-07-8 disrupts cellular microtubule organization, as nocodazole does.

Next, I investigated whether CAS 879127-07-8 disrupts microtubule organization through a direct or indirect action on microtubules. In an *in vitro* microtubule polymerization assay, CAS 879127-07-8 and nocodazole (but not PD153035) inhibited tubulin polymerization in a concentration-dependent manner (Fig. 4B), indicating that CAS 879127-07-8 acts directly on microtubule polymerization. Collectively, these data show that CAS 879127-07-8 has a dual activity: microtubule depolymerization and kinase inhibition.

### Structural insight of dual activity of CAS 879127-07-8

Finally, I investigated the structural basis of the dual activity of CAS 879127-07-8. Models of the complexes between CAS 879127-07-8 and EGFR, and between CAS 879127-07-8 and tubulin, were constructed by docking simulation (Fig. 4C). The simulation predicted that CAS 879127-07-8 docks into an ATP-binding site of EGFR. This prediction is in accordance with a report^27^ in which binding of CAS 879127-07-8 to the ATP-binding site was confirmed biochemically. Notably, for CAS 879127-07-8 to fit into the ATP-binding site, it must adopt an extended structure (Fig. 4C). On the contrary, it must assume a bent configuration to dock in the interface between alpha- and beta-tubulin. CAS 879127-07-8 has seven rotatable arms, which might contribute to its structural flexibility between the extended and bent forms.

## Discussion

In this study, I showed that CAS 879127-07-8, a previously described EGFR inhibitor, also affects microtubule polymerization. Thus, this compound affects two unrelated molecules, both of which are targets of anti-cancer drugs. Mutations of EGFR are frequently observed in various cancers, whereas inhibitors of microtubules are capable of blocking mitosis. As a blocker of mitosis, CAS 879127-07-8 suppressed cell growth in a concentration-dependent manner (Supplementary Fig. S9).

I evaluated the phenotypic effects on EGFR signaling and trafficking using 14 commercially available compounds related to the EGFR signaling pathway. To this end, I subjected microscopic images to high-content screening using automated microscopy and image analysis software. For the image analysis, I defined two intracellular regions, perinuclear and PM, which worked well as reported previously^37^. Subsequently, image analyses were combined using classical statistical methods, such as the KS test, PC analysis, and hierarchical clustering.

As a result of these combined analyses, the 14 compounds were classified into four clusters. The separation of cluster 1 from the other three reflected differences in the PC1 scores, which were heavily influenced by phosphorylated Akt. Half of the compounds examined, including the DMSO control, were classified into the PC1. However, this does not necessarily mean that these compounds have small effects on EGFR signaling. In this study, I selected two major signaling molecules, ERK and Akt, which represent the Ras-MEK-MAPK pathway and PtdIns3K-Akt-mTOR pathway, respectively. Other signaling pathways, including the p38MAPK, PKC, JAK-STAT, and JNK pathways, are also important for cell growth and tumorigenesis. Adoption of these signaling molecules for phenotypic observation in the high-content screen might separate some of the compounds in cluster 1 from the DMSO control.

One major finding in this study was that CAS 879127-07-8 was categorized into cluster 4 together with nocodazole. This was totally unexpected because CAS 879127-07-8 was reported to be a uni-specific inhibitor with only one target among 300 kinases tested. In its extended form, CAS 879127-07-8 binds to an ATP-binding pocket as gefitinib does^27^. The chemical structure of CAS 879127-07-8 consists of three benzenes linked by seven rotatable arms, which may endow CAS 879127-07-8 with structural flexibility. My simulation study predicted that CAS 879127-07-8 binds to the interface between alpha- and beta-tubulin similarly to the manner predicted *in silico* for the microtubule depolymerizer colchicine^54^. Thus, CAS 879127-07-8 could be used as a seed to develop a novel anti-cancer drug that simultaneously inhibits two unrelated proteins, EGFR and microtubules. Because EGFR is one of the major driving forces in cancer metastasis, it is a promising candidate of anti-cancer drugs; consequently, gefitinib and erlotinib are widely used in the treatment of lung cancer^16^,^17^,^18^. Microtubule inhibitors, some of which are used as anti-cancer drugs, can inhibit mitosis^55^. This study showed that CAS 879127-07-8 also inhibits mitosis, as shown in Supplementary Fig. S9. Thus, CAS 879127-07-8 functions as a uni-specific kinase inhibitor and a microtubule depolymerizer. One other molecule, S9, is known to be a dual inhibitor of microtubules and a kinase; such dual-target drugs represent a new strategy for the targeted therapy of cancer^56^.

In summary, I developed a quantitative and statistical system for analyzing microscopic images that reflect the effects of various inhibitors on signal transduction and vesicle trafficking of EGFR. I demonstrated that a highly specific kinase inhibitor, CAS 879127-07-8, also functions as an inhibitor of microtubules as well. Thus, this system appears to be a powerful tool for discovering unexpected properties of known inhibitors. In the future, this system should be applicable to studies of other RTKs, as well as the relationship between intracellular signaling and vesicle trafficking.

## Methods

### Cells and reagents

A549 GFP-EGFR cells, which express GFP-tagged endogenous EGFR protein, were purchased from Sigma (CLL1141) and maintained at 37 °C in DMEM containing 10% FBS and antibiotic-antimycotic solution (A5955, Sigma). A549 cells and the lung cancer cell panel were purchased from the RIKEN BioResource Center and ATCC, respectively, and maintained in RPMI-1640 containing 10% FBS (except for H1573, whose medium contains 5% FBS) and antibiotic-antimycotic solution. Recombinant EGF and human transferrin were purchased from R&D Systems. Alexa Fluor 647-conjugated EGF, Alexa Fluor 647-conjugated human transferrin, and Hoechst 33342 were from Invitrogen. Rabbit anti-phosphorylated ERK monoclonal antibody and rabbit anti-phosphorylated Akt monoclonal antibody were purchased from Cell Signaling Technologies. Mouse anti-PtdIns(4)P monoclonal antibody and mouse anti-PtdIns(4,5)P_2_ monoclonal antibody were purchased from Echelon. Rabbit anti-GST polyclonal antibody was purchased from Santa Cruz Biotechnology. Alexa Fluor-conjugated secondary antibodies were purchased from Invitrogen. All antibodies were preserved at −30 °C after addition of an equal volume of glycerol. Compounds were purchased as an EGFR inhibitor panel (Merck Millipore) except for nocodazole (Sigma).

### Recombinant proteins

pGEX-HrsFYVE was transformed into BL21 (DE3), and recombinant protein was induced by incubation with 0.1 mM IPTG for 1 h at 37 °C. Cells were lysed for 30 min at 4 °C using B-PER bacterial protein extraction reagent (Pierce) containing Complete^TM^ protease inhibitor cocktail (Roche). Cells were clarified by centrifugation (1 min at 15,000 × *g*), and the resultant supernatants were rocked with glutathione-Sepharose 4B (Amersham) for 1 h. Beads were collected by centrifugation (5 min at 600 × *g*), and then washed three times with B-PER. GST-HrsFYVE was eluted from the beads with B-PER containing 20 mM glutathione. Eluted proteins were frozen and used for experiments.

### Inhibitor treatment and EGF stimulation

Cells were seeded on Edge-plates (Thermo Scientific), serum-starved for 6 h by replacing the medium with DMEM containing 0.1% BSA, and stimulated with 100 ng/ml EGF (R&D Systems). One hour before EGF stimulation, cells were treated with various compounds at the indicated concentrations: Akt Inhibitor VIII (200 nM)^57^, PD153035 (100 nM)^58^, CAS 879127-07-8 (10 μM)^27^, Et-18-OCH3 (5 μg/ml)^59^, JNK inhibitor II (1 μM)^60^, LY294002 (10 μM)^57^, PD98059 (50 μM)^61^, PD168393 (100 nM)^62^, PP2 (100 nM)^63^, rapamycin (10 nM)^64^, SB253080 (10 μM)^60^, AG490 (40 μM)^65^, ZM336372 (1 μM)^66^, and nocodazole (50 μM)^67^. To observe internalized EGF, Alexa Fluor 647-conjugated EGF was used for initial internalization, and chased after 5 min with unlabeled EGF. To observe internalized transferrin, Alexa Fluor 647-conjugated transferrin and unlabeled EGF were internalized for 5 min, and then chased with unlabeled human transferrin (R&D Systems) and EGF. After the indicated intervals, cells were fixed by addition of an equal volume of 4% paraformaldehyde (09154-85, Nacalai Tesque) for 15 min, followed by washing in PBS.

### Immunofluorescence

Fixed cells were subsequently processed for immunostaining. To stain phosphorylated ERK, aldehyde-fixed cells were further incubated with ice-cold methanol for 10 min at −30 °C, washed with PBS, and incubated in blocking buffer (1% BSA in PBS) containing 0.3% Triton X-100 (TX100) for 1 h at RT. To stain phosphorylated Akt, fixed cells were directly incubated in blocking buffer containing TX100. To stain phosphoinositides, fixed cells were permeabilized with 0.01% digitonin in PBS for 5 min at RT, washed with PBS, and incubated in blocking buffer without TX100 for 1 h at RT. To observe PtdIns(3)P, recombinant GST-Hrs FYVE protein was added to the blocking buffer, and after incubation for 1 h at RT, cells were washed with PBS. Blocked cells were incubated with the following primary antibodies for 1 h at RT: anti-pERK (1:1000), anti-pAkt (1:2000), anti-PtdIns(4)P (1:2000), anti-PtdIns(4,5)P2 (1:1000), or anti-GST (1:200). Cells were washed with PBS two times and subsequently incubated for 1 h at RT with the appropriate secondary antibodies and Hoechst 33432. Cells were washed twice with PBS, fixed with 2% paraformaldehyde for 5 min at RT, washed with PBS, and stored at 4 °C.

### Image acquisition and analysis

Stained cells were photographed using a cell image analyzer (CellInsight, Thermo Scientific) equipped with a 20× objective lens. Hoechst and GFP signals were used in all marker sets to define the ‘nucleus’ and ‘cell’ regions, respectively. In each well, 36 fields were photographed. Image analysis was performed using CellProfiler (Broad Institute), and all images were processed for illumination correction. Using the primary object ‘nucleus’ and GFP-EGFR signals, the cell outline (‘cell’) was identified by the ‘propagation’ implemented in CellProfiler. ‘Cytoplasm’ was defined by the subtraction of ‘nucleus’ from ‘cell’. The ‘nucleus’ and ‘cell’ regions were expanded and shrunk by 5 and 7 pixels, respectively, and the resultant regions were named ‘perinuclear’ and ‘PM’, respectively. These regions of interest (ROIs) were used to quantitate each image feature. The signals of some fluorescent signals were converted into objects to identify ‘endosomes’ using the Top-Hat filter implemented in the ‘enhance’ module in CellProfiler. Co-localization was also evaluated using these objects. Descriptors used in this study are listed in Supplementary Table S1.

### Data processing and statistical analysis

Data was stored in a local MySQL server and processed for statistical analysis using MatLab (Mathworks). Selected image features (descriptors) of each compound were compared with control (DMSO-treated) cells using the one-sided two-sample KS test. Addition of a sign to KS statistics was performed according to the method of Perlman *et al.*^24^. Signed KS statistics were divided by the corresponding control standard deviation (Std), which was estimated by bootstrap simulation with replacement (n = 1000) to yield a standardized Z-score. When the correlation coefficient of the Z-score between two independent experiments was above 0.4, the descriptor was accepted for subsequent analysis. A total of 570 Z-scores were accepted, and then subjected to principal component analysis (PCA). Seven PCs explained over 95% of the variance, and were used for subsequent hierarchical clustering using the Euclid distance and Ward method.

### Bioinformatics

Known targets of compounds were compiled using information from the ChEMBL database (EMBL-EBI) and selected according to the following criteria: 1) the IC_50_ is below the concentration used in this study; 2) the target is indicated as a ‘single protein’; and 3) the entry was curated by an ‘expert’ (not ‘autocuration’). Target prediction was performed using SwissTargetPrediction (SWISS-SBI). Docking simulation was performed using CLC Drug Discovery Workbench 2.1 (Qiagen).

## Additional Information

**How to cite this article**: Tanabe, K. Image-based compound profiling reveals a dual inhibitor of tyrosine kinase and microtubule polymerization. *Sci. Rep.* **6**, 25095; doi: 10.1038/srep25095 (2016).

## Supplementary Material

Supplementary Information

## References

[b1] Feng Y., Mitchison T. J., Bender A., Young D. W. & Tallarico J. A. Multi-parameter phenotypic profiling: using cellular effects to characterize small-molecule compounds. Nat. Rev. Drug Discov. 8, 567–78 (2009). 19568283 10.1038/nrd2876

[b2] Zhang J., Yang P. L. & Gray N. S. Targeting cancer with small molecule kinase inhibitors. Nat. Rev. Cancer 9, 28–39 (2009). 19104514 10.1038/nrc2559 PMC12406740

[b3] Johannessen C. M., Clemons P. A. & Wagner B. K. Integrating phenotypic small-molecule profiling and human genetics: the next phase in drug discovery. Trends Genet. 31, 16–23 (2014). 25498789 10.1016/j.tig.2014.11.002 PMC4281276

[b4] Anastassiadis T., Deacon S. W., Devarajan K., Ma H. & Peterson J. R. Comprehensive assay of kinase catalytic activity reveals features of kinase inhibitor selectivity. Nat. Biotechnol. 29, 1039–45 (2011). 22037377 10.1038/nbt.2017 PMC3230241

[b5] Cohen P. Guidelines for the effective use of chemical inhibitors of protein function to understand their roles in cell regulation. Biochem. J. 425, 53–4 (2010). 20001962 10.1042/BJ20091428

[b6] Ljosa V. *et al.* Comparison of methods for image-based profiling of cellular morphological responses to small-molecule treatment. J. Biomol. Screen. 18, 1321–9 (2013). 24045582 10.1177/1087057113503553 PMC3884769

[b7] Tanaka M. *et al.* An unbiased cell morphology-based screen for new, biologically active small molecules. PLoS Biol. 3, e128 (2005). 15799708 10.1371/journal.pbio.0030128 PMC1073692

[b8] Bain J., McLauchlan H., Elliott M. & Cohen P. The specificities of protein kinase inhibitors: an update. Biochem. J. 371, 199–204 (2003). 12534346 10.1042/BJ20021535 PMC1223271

[b9] Bain J. *et al.* The selectivity of protein kinase inhibitors: a further update. Biochem. J. 408, 297–315 (2007). 17850214 10.1042/BJ20070797 PMC2267365

[b10] Davies S., Reddy H., Caivano M. & Cohen P. Specificity and mechanism of action of some commonly used protein kinase inhibitors. Biochem. J 105, 95–105 (2000). 10998351 10.1042/0264-6021:3510095 PMC1221339

[b11] Ashburn T. T. & Thor K. B. Drug repositioning: identifying and developing new uses for existing drugs. Nat. Rev. Drug Discov. 3, 673–83 (2004). 15286734 10.1038/nrd1468

[b12] Iskar M., Zeller G., Zhao X.-M., van Noort V. & Bork P. Drug discovery in the age of systems biology: the rise of computational approaches for data integration. Curr. Opin. Biotechnol. 23, 609–16 (2012). 22153034 10.1016/j.copbio.2011.11.010

[b13] Ciardiello F. & Tortora G. EGFR antagonists in cancer treatment. N. Engl. J. Med. 358, 1160–74 (2008). 18337605 10.1056/NEJMra0707704

[b14] Antczak C., Mahida J. P., Bhinder B., Calder P. A. & Djaballah H. A high-content biosensor-based screen identifies cell-permeable activators and inhibitors of EGFR function: implications in drug discovery. J. Biomol. Screen. 17, 885–99 (2012). 22573732 10.1177/1087057112446174 PMC3615554

[b15] Mellman I. & Yarden Y. Endocytosis and cancer. Cold Spring Harb. Perspect. Biol. 5, 1–24 (2013). 10.1101/cshperspect.a016949 PMC3839607 24296170

[b16] Song H. *et al.* Functional characterization of pulmonary neuroendocrine cells in lung development, injury, and tumorigenesis. Proc. Natl. Acad. Sci. 109, 17531–17536 (2012). 23047698 10.1073/pnas.1207238109 PMC3491514

[b17] Sequist L. V. *et al.* Genotypic and histological evolution of lung cancers acquiring resistance to EGFR inhibitors. Sci. Transl. Med. 3, 75ra26 (2011). 10.1126/scitranslmed.3002003 PMC3132801 21430269

[b18] Tanoue L. T. EGFR Mutations in Lung Cancer: Correlation With Clinical Response to Gefitinib Therapy. Yearb. Pulm. Dis. 2006, 112–114 (2006).

[b19] Marks J. L. *et al.* Novel MEK1 mutation identified by mutational analysis of epidermal growth factor receptor signaling pathway genes in lung adenocarcinoma. Cancer Res. 68, 5524–5528 (2008). 18632602 10.1158/0008-5472.CAN-08-0099 PMC2586155

[b20] Mascaux C. *et al.* The role of RAS oncogene in survival of patients with lung cancer: a systematic review of the literature with meta-analysis. Br. J. Cancer 92, 131–139 (2005). 15597105 10.1038/sj.bjc.6602258 PMC2361730

[b21] Kawano O. *et al.* PIK3CA mutation status in Japanese lung cancer patients. Lung Cancer 54, 209–15 (2006). 16930767 10.1016/j.lungcan.2006.07.006

[b22] Lill N. L. & Sever N. I. Where EGF receptors transmit their signals. Sci. Signal. 5, pe41 (2012). 23012653 10.1126/scisignal.2003341 PMC3507515

[b23] Tomas A., Futter C. E. & Eden E. R. EGF receptor trafficking: consequences for signaling and cancer. Trends Cell Biol. 24, 1–9 (2013). 10.1016/j.tcb.2013.11.002 PMC3884125 24295852

[b24] Perlman Z. E. *et al.* Multidimensional drug profiling by automated microscopy. Science 306, 1194–8 (2004). 15539606 10.1126/science.1100709

[b25] Young D. W. *et al.* Integrating high-content screening and ligand-target prediction to identify mechanism of action. Nat. Chem. Biol. 4, 59–68 (2008). 18066055 10.1038/nchembio.2007.53

[b26] Loo L.-H., Wu L. F. & Altschuler S. J. Image-based multivariate profiling of drug responses from single cells. 4, 445–53 (2007). 10.1038/nmeth1032 17401369

[b27] Zhang Q. *et al.* Discovery of EGFR selective 4,6-disubstituted pyrimidines from a combinatorial kinase-directed heterocycle library. J. Am. Chem. Soc. 128, 2182–3 (2006). 16478150 10.1021/ja0567485

[b28] Anastassiadis T., Deacon S. & Devarajan K. Comprehensive assay of kinase catalytic activity reveals features of kinase inhibitor selectivity. Nat. Biotechnol. 29, 1039–1045 (2011). 22037377 10.1038/nbt.2017 PMC3230241

[b29] Sigismund S. *et al.* Clathrin-mediated internalization is essential for sustained EGFR signaling but dispensable for degradation. Dev. Cell 15, 209–19 (2008). 18694561 10.1016/j.devcel.2008.06.012

[b30] Sigismund S. *et al.* Clathrin-independent endocytosis of ubiquitinated cargos. Proc. Natl. Acad. Sci. USA 102, 2760–2765 (2005). 15701692 10.1073/pnas.0409817102 PMC549482

[b31] Martin T. F. PI(4,5)P(2) regulation of surface membrane traffic. Curr. Opin. Cell Biol. 13, 493–499 (2001). 11454457 10.1016/s0955-0674(00)00241-6

[b32] Chu K. M. E., Minogue S., Hsuan J. J. & Waugh M. G. Differential effects of the phosphatidylinositol 4-kinases, PI4KIIα and PI4KIIIβ, on Akt activation and apoptosis. Cell Death Dis. 1, e106 (2010). 21218173 10.1038/cddis.2010.84 PMC3015391

[b33] Schink K. O., Raiborg C. & Stenmark H. Phosphatidylinositol 3-phosphate, a lipid that regulates membrane dynamics, protein sorting and cell signalling. BioEssays 35, 900–912 (2013). 23881848 10.1002/bies.201300064

[b34] Mayle K. M., Le A. M. & Kamei D. T. The intracellular trafficking pathway of transferrin. Biochim. Biophys. Acta - Gen. Subj. 1820, 264–281 (2012). 10.1016/j.bbagen.2011.09.009 PMC3288267 21968002

[b35] Mesaki K., Tanabe K., Obayashi M., Oe N. & Takei K. Fission of tubular endosomes triggers endosomal acidification and movement. PLoS One 6, e19764 (2011). 21572956 10.1371/journal.pone.0019764 PMC3091875

[b36] Ohashi E. *et al.* Receptor Sorting within Endosomal Trafficking Pathway Is Facilitated by Dynamic Actin Filaments. PLoS One 6, e19942 (2011). 21625493 10.1371/journal.pone.0019942 PMC3098849

[b37] Liberali P., Snijder B. & Pelkmans L. A Hierarchical Map of Regulatory Genetic Interactions in Membrane Trafficking. Cell 157, 1473–1487 (2014). 24906158 10.1016/j.cell.2014.04.029

[b38] Minogue S. *et al.* Phosphatidylinositol 4-kinase is required for endosomal trafficking and degradation of the EGF receptor. J Cell Sci 119, 571–581 (2006). 16443754 10.1242/jcs.02752

[b39] Mendoza M. C., Er E. E. & Blenis J. The Ras-ERK and PI3K-mTOR pathways: cross-talk and compensation. Trends Biochem. Sci. 36, 320–8 (2011). 21531565 10.1016/j.tibs.2011.03.006 PMC3112285

[b40] Young T. Proof without prejudice: use of the Kolmogorov-Smirnov test for the analysis of histograms from flow systems and other sources. J. Histochem. Cytochem. 25, 935–941 (1977). 894009 10.1177/25.7.894009

[b41] Lampariello F. On the use of the Kolmogorov-Smirnov statistical test for immunofluorescence histogram comparison. Cytometry 39, 179–88 (2000). 10685074 10.1002/(SICI)1097-0320(20000301)39:3<179::AID-CYTO2>3.0.CO;2-I

[b42] Balla T. Phosphoinositides: tiny lipids with giant impact on cell regulation. Physiol. Rev. 93, 1019–137 (2013). 23899561 10.1152/physrev.00028.2012 PMC3962547

[b43] Lali F. V., Hunt a E., Turner S. J. & Foxwell B. M. The pyridinyl imidazole inhibitor SB203580 blocks phosphoinositide-dependent protein kinase activity, protein kinase B phosphorylation, and retinoblastoma hyperphosphorylation in interleukin-2-stimulated T cells independently of p38 mitogen-activated prot. J. Biol. Chem. 275, 7395–402 (2000). 10702313 10.1074/jbc.275.10.7395

[b44] Alam M. M. *et al.* Synthesis, characterization and Akt phosphorylation inhibitory activity of cyclopentanecarboxylate-substituted alkylphosphocholines. Bioorg. Med. Chem. 21, 2018–24 (2013). 23415083 10.1016/j.bmc.2013.01.010

[b45] Vlahos C. J., Matter W. F., Hui K. Y. & Brown R. F. A specific inhibitor of phosphatidylinositol 3-kinase, 2-(4-morpholinyl)-8-phenyl-4H-1-benzopyran-4-one (LY294002). J. Biol. Chem. 269, 5241–5248 (1994). 8106507

[b46] Barnett S. F. *et al.* Identification and characterization of pleckstrin-homology-domain-dependent and isoenzyme-specific Akt inhibitors. Biochem. J. 385, 399–408 (2005). 15456405 10.1042/BJ20041140 PMC1134710

[b47] Calleja V., Laguerre M., Parker P. J. & Larijani B. Role of a novel PH-kinase domain interface in PKB/Akt regulation: structural mechanism for allosteric inhibition. PLoS Biol. 7, e17 (2009). 19166270 10.1371/journal.pbio.1000017 PMC2628406

[b48] Bos M. *et al.* PD153035, a tyrosine kinase inhibitor, prevents epidermal growth factor receptor activation and inhibits growth of cancer cells in a receptor number-dependent manner. Clin. Cancer Res. 3, 2099–106 (1997). 9815602

[b49] Pu Y.-S. *et al.* Epidermal growth factor receptor inhibitor (PD168393) potentiates cytotoxic effects of paclitaxel against androgen-independent prostate cancer cells. Biochem. Pharmacol. 71, 751–60 (2006). 16413505 10.1016/j.bcp.2005.12.009

[b50] DeBrabander M. J., Van deVeire R. M. L., Aerts F. E. M., Borgers M. & Janssen P. a J. The effects of methyl [5-(2-thienylcarbonyl)-1H-benzimidazol-2-y] carbamate, (R 17934; NSC 238159), a new synhetic antirumoral drug interfering with microtubules on mammalian cells cultured *in vivo*. Cancer Res. 36, 905 (1976). 766963

[b51] Park H., Hong S. & Hong S. Nocodazole is a high-affinity ligand for the cancer-related kinases ABL, c-KIT, BRAF, and MEK. Chem Med Chem 7, 53–6 (2012). 22002881 10.1002/cmdc.201100410

[b52] Sorkin A. & Goh L. K. Endocytosis and intracellular trafficking of ErbBs. Exp. Cell Res. 315, 683–696 (2009). 19278030 10.1016/j.yexcr.2008.07.029 PMC2605728

[b53] Marek L. *et al.* Fibroblast Growth Factor (FGF) and FGF Receptor-Mediated Autocrine Signaling in Non – Small-Cell Lung Cancer Cells. Mol. Pharmacol. 75, 196–207 (2009). 18849352 10.1124/mol.108.049544 PMC2669785

[b54] Ravelli R., Gigant B., Curmi P. & Jourdain I. Insight into tubulin regulation from a complex with colchicine and a stathmin-like domain. Nature 428, 198–202 (2004). 15014504 10.1038/nature02393

[b55] Jordan M. & Wilson L. Microtubules as a target for Anticancer drugs. Nat. Rev. Cancer 4, 253–265 (2004). 15057285 10.1038/nrc1317

[b56] Zhang C. *et al.* S9, a novel anticancer agent, exerts its anti-proliferative activity by interfering with both PI3K-Akt-mTOR signaling and microtubule cytoskeleton. PLoS One 4, e4881 (2009). 19293927 10.1371/journal.pone.0004881 PMC2654064

[b57] Choi E. J. *et al.* Targeting epidermal growth factor receptor-associated signaling pathways in non-small cell lung cancer cells: implication in radiation response. Mol. cancer Res. 8, 1027–36 (2010). 20587532 10.1158/1541-7786.MCR-09-0507

[b58] Lichtner R. B., Menrad A., Sommer A., Klar U. & Schneider M. R. Signaling-inactive Epidermal Growth Factor Receptor/Ligand Complexes in Intact Carcinoma Cells by Quinazoline Tyrosine Kinase Inhibitors. Cancer Res. 61, 5790–5795 (2001). 11479217

[b59] Lu X. & Arthur G. The Differential Susceptibility of A427 and A549 Cell Lines to the Growth-inhibitory Effects of ET-18-OCH3 Does Not Correlate with the Relative Effects of the Alkyl-lysophospholipid on the Incorporation of Fatty Acids into Cellular Phospholipids. Cancer Res. 52, 2813–2817 (1992). 1581895

[b60] Kurinna S. M., Tsao C. C., Nica A. F., Jiffar T. & Ruvolo P. P. Ceramide promotes apoptosis in lung cancer-derived A549 cells by a mechanism involving c-Jun NH2-terminal kinase. Cancer Res. 64, 7852–6 (2004). 15520191 10.1158/0008-5472.CAN-04-1552

[b61] Coogan A. N., O’Leary D. M. & O’Connor J. J. P42/44 MAP Kinase Inhibitor PD98059 Attenuates Multiple Forms of Synaptic Plasticity in Rat Dentate Gyrus *In Vitro*. J Neurophysiol 81, 103–110 (1999). 9914271 10.1152/jn.1999.81.1.103

[b62] Koprivica V. *et al.* EGFR activation mediates inhibition of axon regeneration by myelin and chondroitin sulfate proteoglycans. Science 310, 106–10 (2005). 16210539 10.1126/science.1115462

[b63] Dittmann K., Mayer C., Kehlbach R. & Rodemann H. P. Radiation-induced caveolin-1 associated EGFR internalization is linked with nuclear EGFR transport and activation of DNA-PK. Mol. Cancer 7, 69 (2008). 18789131 10.1186/1476-4598-7-69 PMC2546440

[b64] Wang X. *et al.* Inhibition of mammalian target of rapamycin induces phosphatidylinositol 3-kinase-dependent and Mnk-mediated eukaryotic translation initiation factor 4E phosphorylation. Mol. Cell. Biol. 27, 7405–13 (2007). 17724079 10.1128/MCB.00760-07 PMC2169067

[b65] Huang W.-L. *et al.* Signal transducer and activator of transcription 3 activation up-regulates interleukin-6 autocrine production: a biochemical and genetic study of established cancer cell lines and clinical isolated human cancer cells. Mol. Cancer 9, 309 (2010). 21122157 10.1186/1476-4598-9-309 PMC3027602

[b66] Carnahan J. *et al.* Selective and potent Raf inhibitors paradoxically stimulate normal cell proliferation and tumor growth. Mol. Cancer Ther. 9, 2399–410 (2010). 20663930 10.1158/1535-7163.MCT-10-0181

[b67] Lehmann M. J., Sherer N. M., Marks C. B., Pypaert M. & Mothes W. Actin- and myosin-driven movement of viruses along filopodia precedes their entry into cells. J. Cell Biol. 170, 317–25 (2005). 16027225 10.1083/jcb.200503059 PMC2171413

