# Supplementary material for: Image-based compound profiling reveals a dual inhibitor of tyrosine kinase and microtubule polymerization
Source: Sci Rep. 2016 Apr 27;6:25095. doi: 10.1038/srep25095 (PMC4846875; doi:10.1038/srep25095)
Supplement: Supplementary Information [file srep25095-s1.pdf]

Image-based compound profiling reveals a dual inhibitor of tyrosine kinase and microtubule polymerization

Kenji Tanabe*

<u>Supplementary information</u>

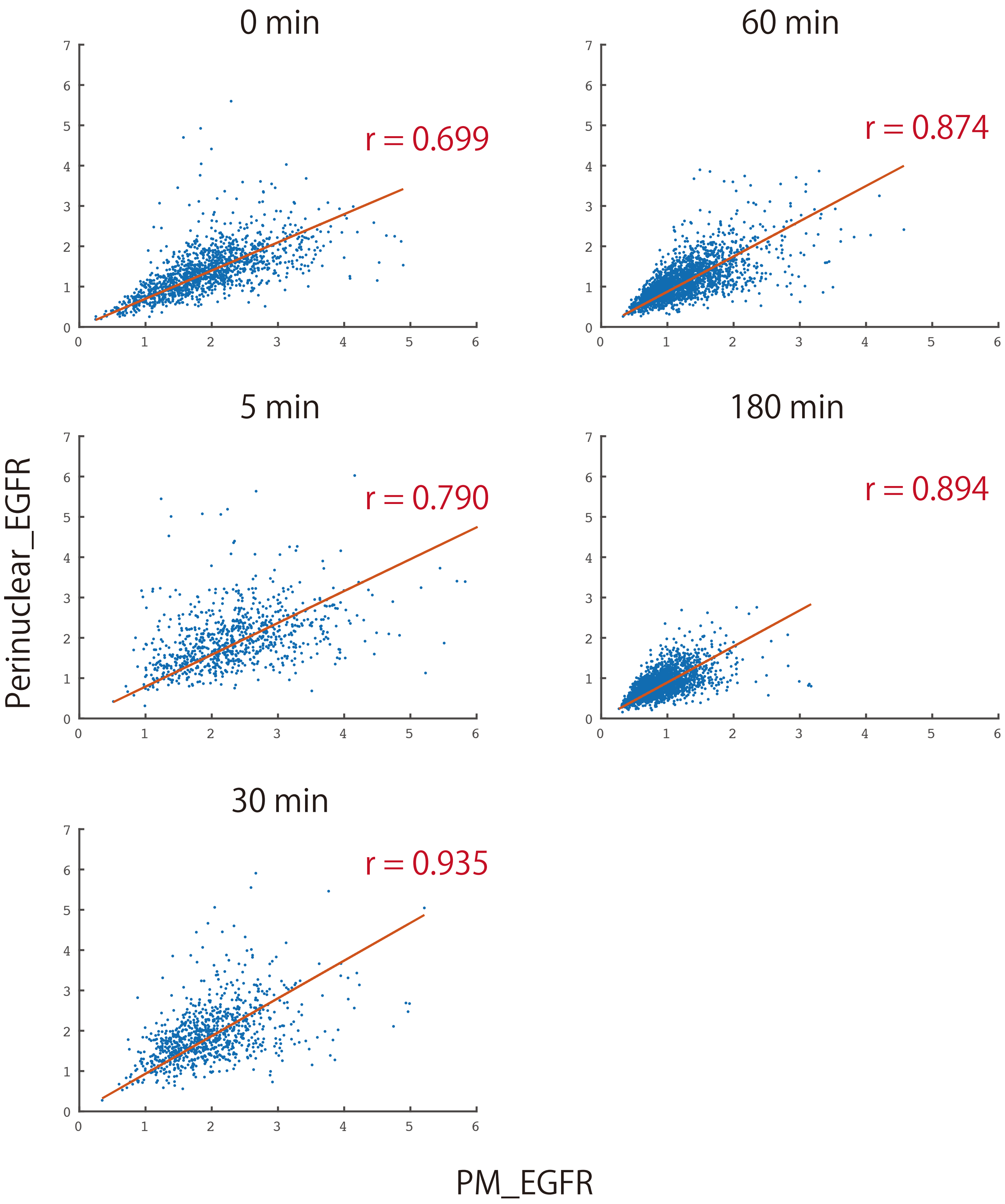

Supplementary Figure S1. Quantitative analysis of EGFR translocation following EGF stimulation. Integrated intensity of GFP-EGFR from the perinuclear or PM region was plotted, and regression coefficients (r) without constant were calculated. Note that r increased after EGF stimulation, reaching a peak at 30 min, suggesting that EGFR was moved toward the perinuclear area following EGF stimulation.

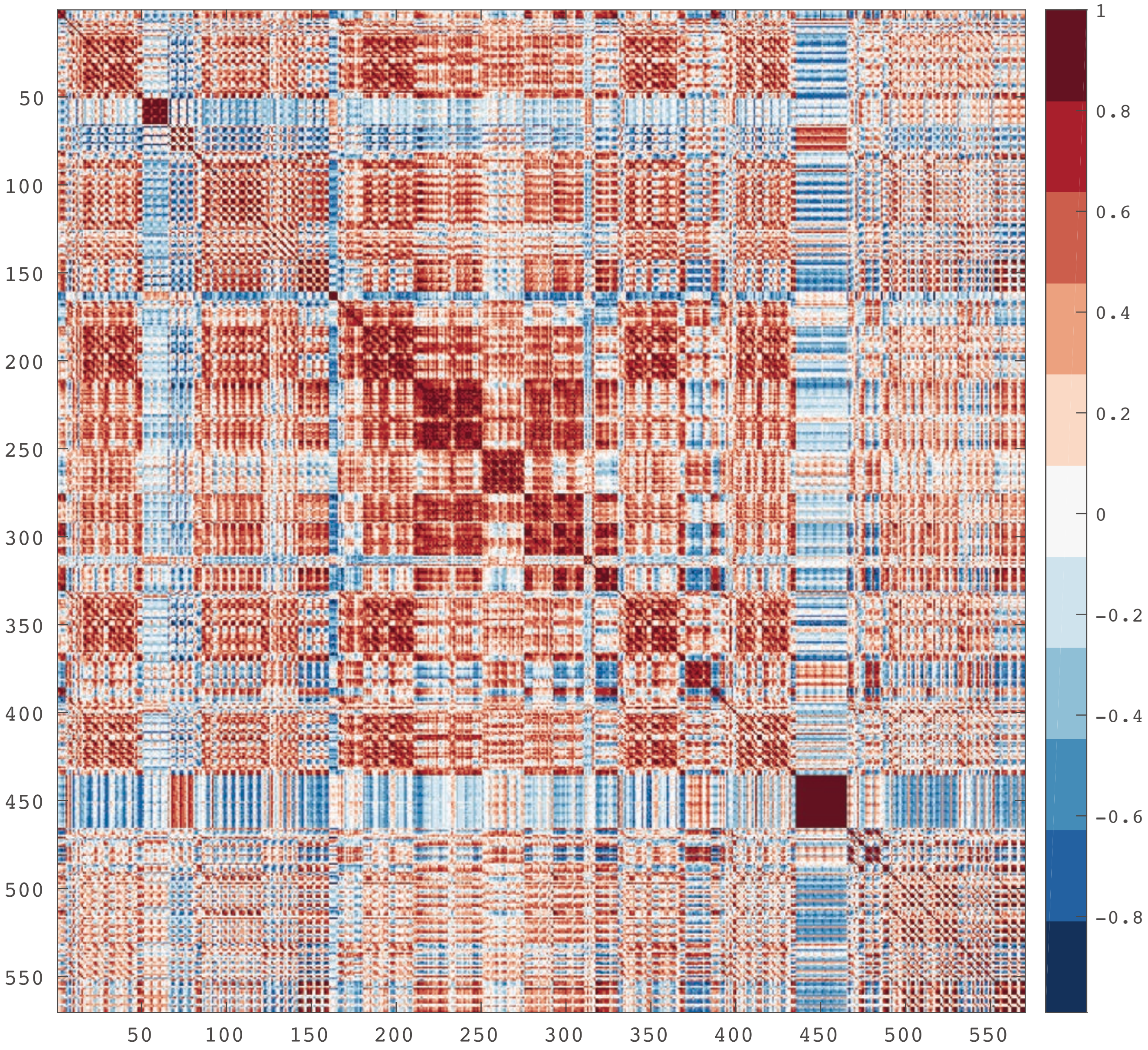

Supplementary Figure S2. Correlation matrix among descriptors used in PCA analysis.
Correlation among 570 descriptors used in PCA analysis were visualized by correlation matrix.

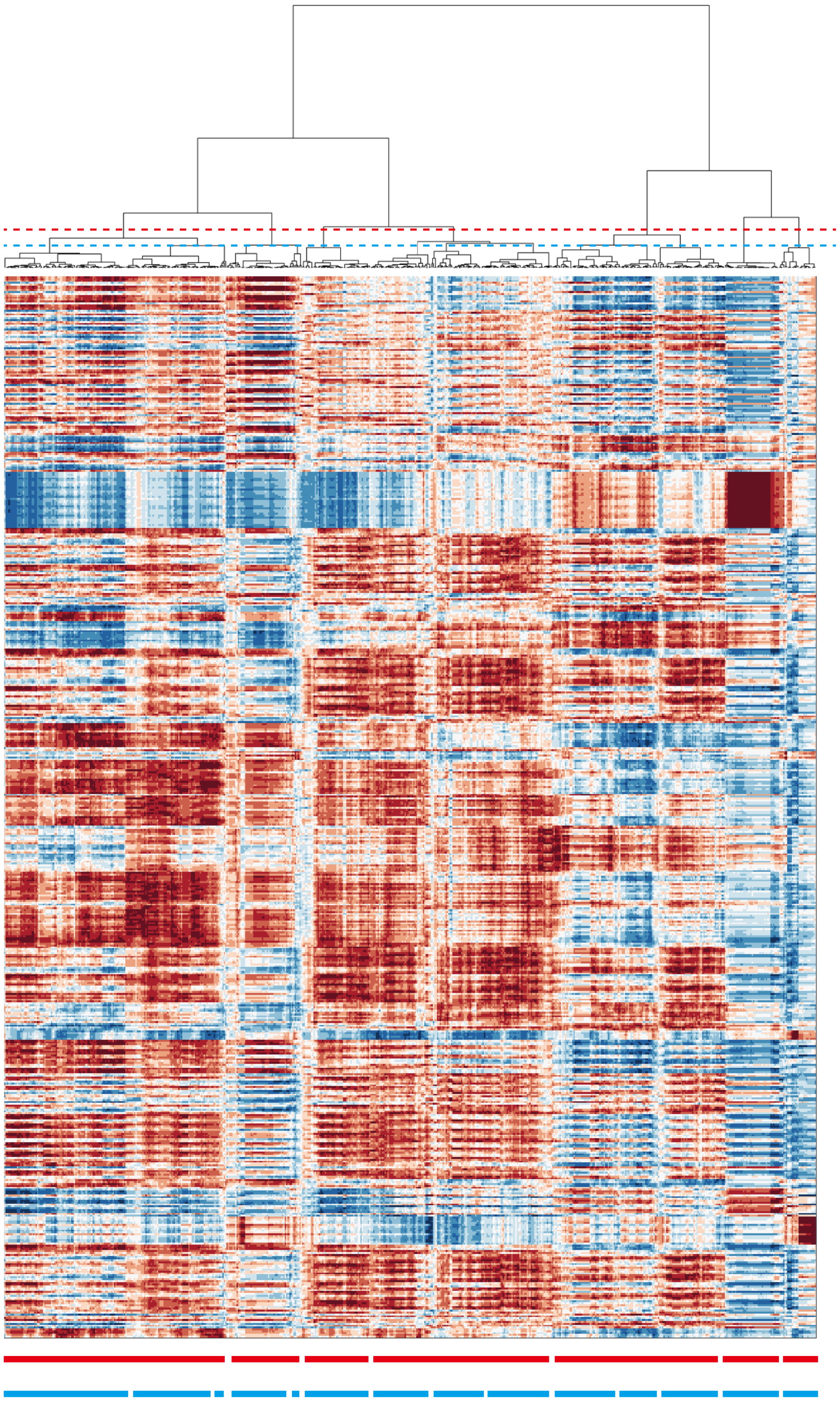

Supplementary Figure S3. Hierarchical clustering of descriptors using correlation coefficients. Descriptors were processed for hierarchical clustering based on their correlation coefficients (supplementary fig. S2). Clustering was performed using the cosine distance and Ward method. Red and blue dotted line show the existence of seven fourteen clusters, respectively.

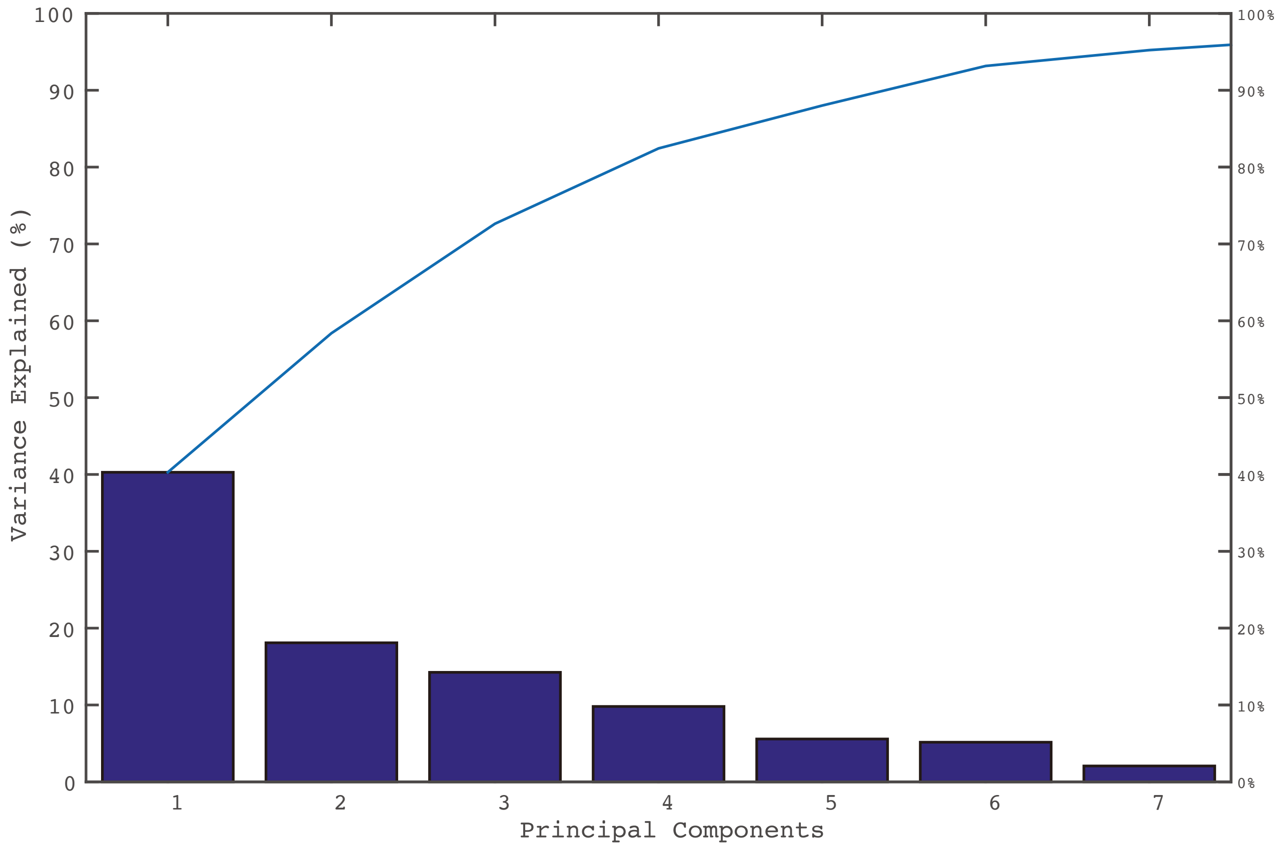

Supplementary Figure S4. Seven principal components explained more than 95% of variance in ~570 descriptors. Bar plots show the variance explained by each principal component, and the line chart indicates cumulative variance.

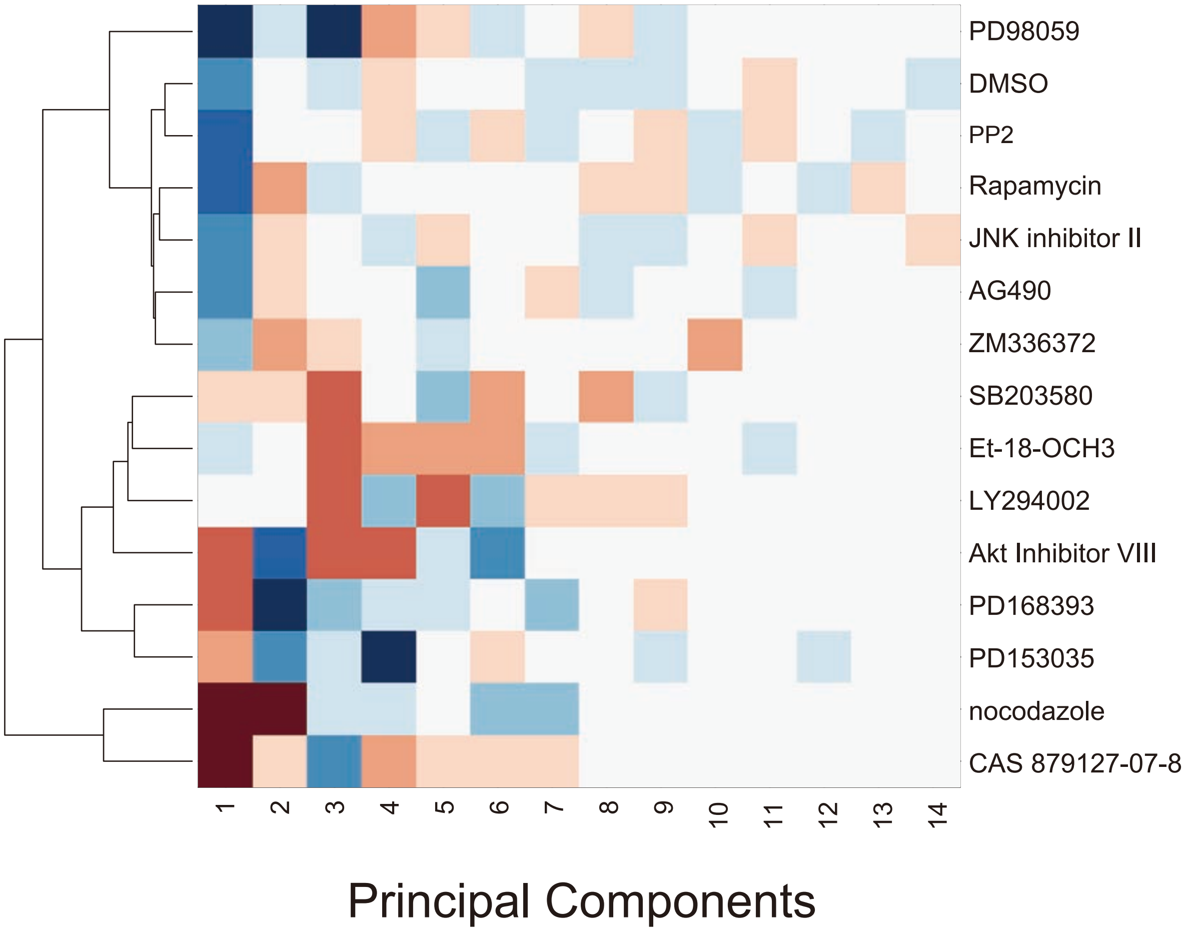

Supplementary Figure S5. Hierarchical clustering using all fourteen principal components (PCs). All PCs, which explains all variance in Z-score used in this analysis, was used for hierarichical clustering using the Euclid distance and Ward method. This clustering is similar to the results from seven significant PCs (Figure 3B).

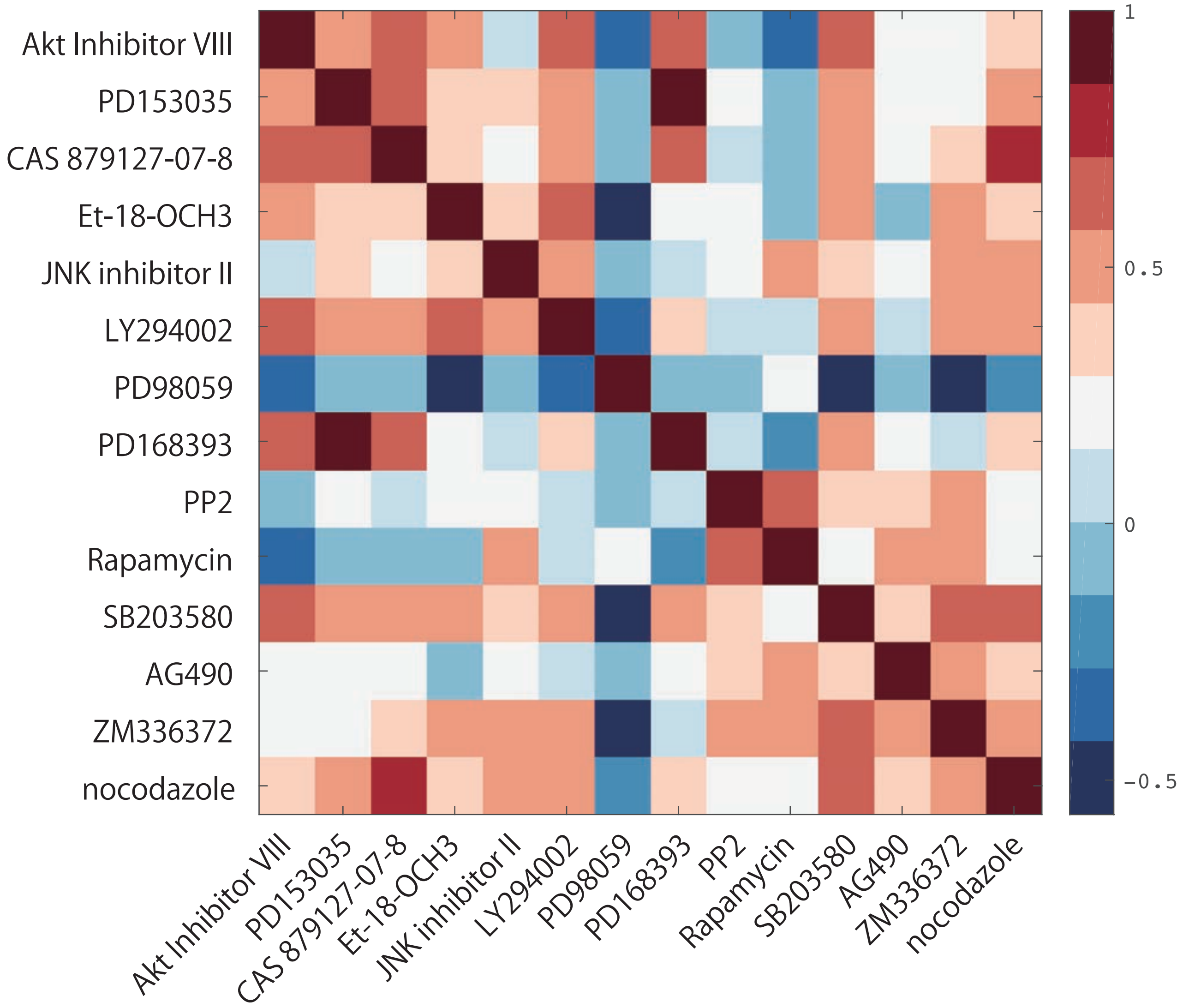

Supplementary Figure S6. Correlation matrix among compounds tested.

Correlation among fourteen compounds were visualized by correlation matrix. Note to that CAS 879127-07-8 between nocodazole showed the highest correlation coefficients.

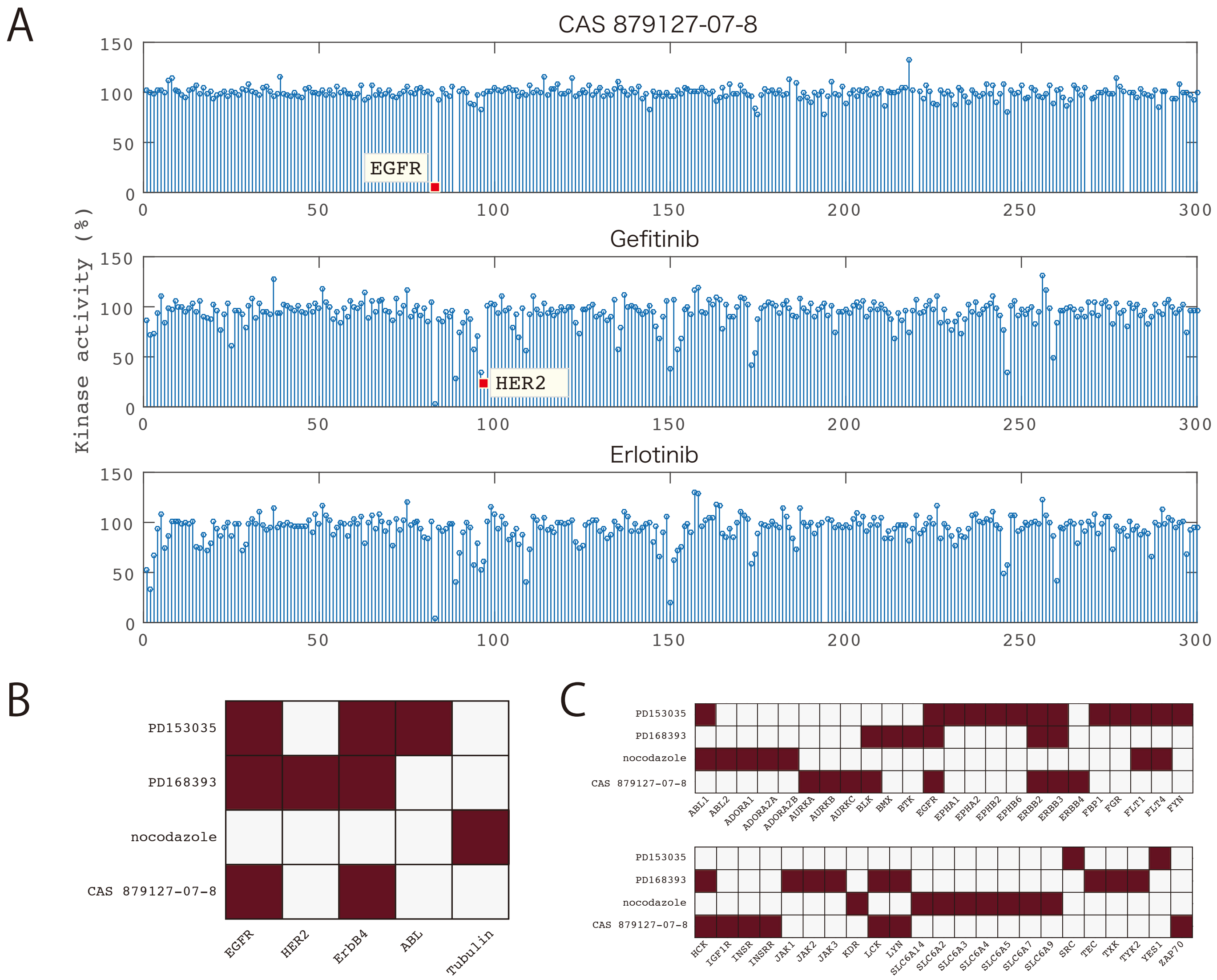

Supplementary Figure S7. No common targets between CAS 879127-07-8 and nocodazole. (A) Inhibitory effects against ~300 kinases in vitro. Data were obtained from Anastassiadis et al., (2011).. Note that CAS 879127-07-8 is uni-specific and inhibits only EGFR. (B) Targets listed in the ChEMBL database, restricted to only those proteins for which the IC50 was below the final concentration used in this study. (C) Targets predicted using SwissTargetPrediction at SWISS-SBI. Only ten candidates for each compound were picked up and summarized.

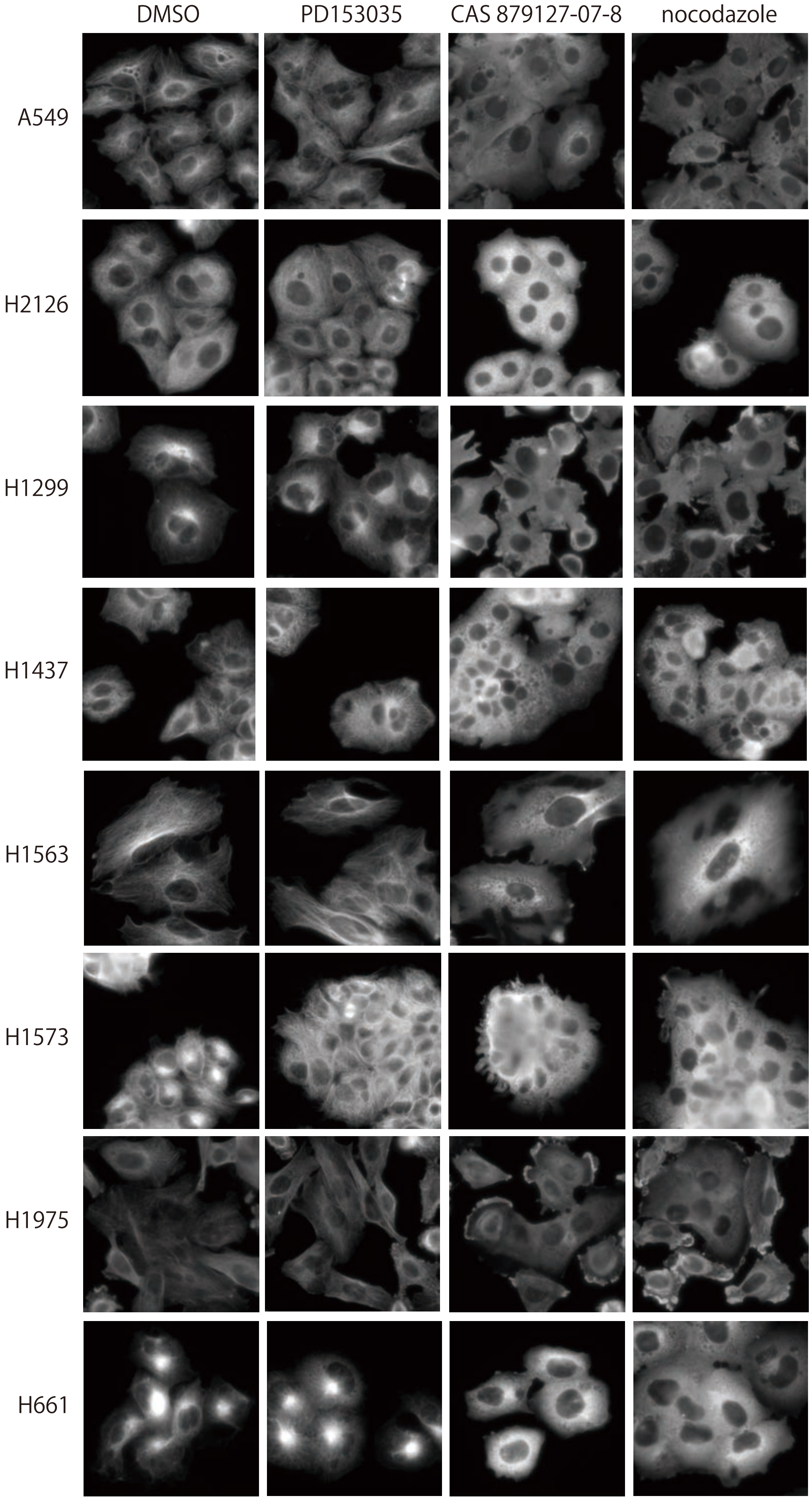

Supplementary Figure S8. Microtubule disruption by CAS 879127-07-8 was observed in various cell lines. Seven lung cancer cell lines were treated with DMSO, PD153035, CAS 879127-07-8, or nocodazole for 1 h, fixed, and immunostained using anti-α-tubulin. Both CAS 879127-07-8 and nocodazole disrupted the radial and fibrous distribution of microtubules observed in DMSO- or PD153035-treated cells.

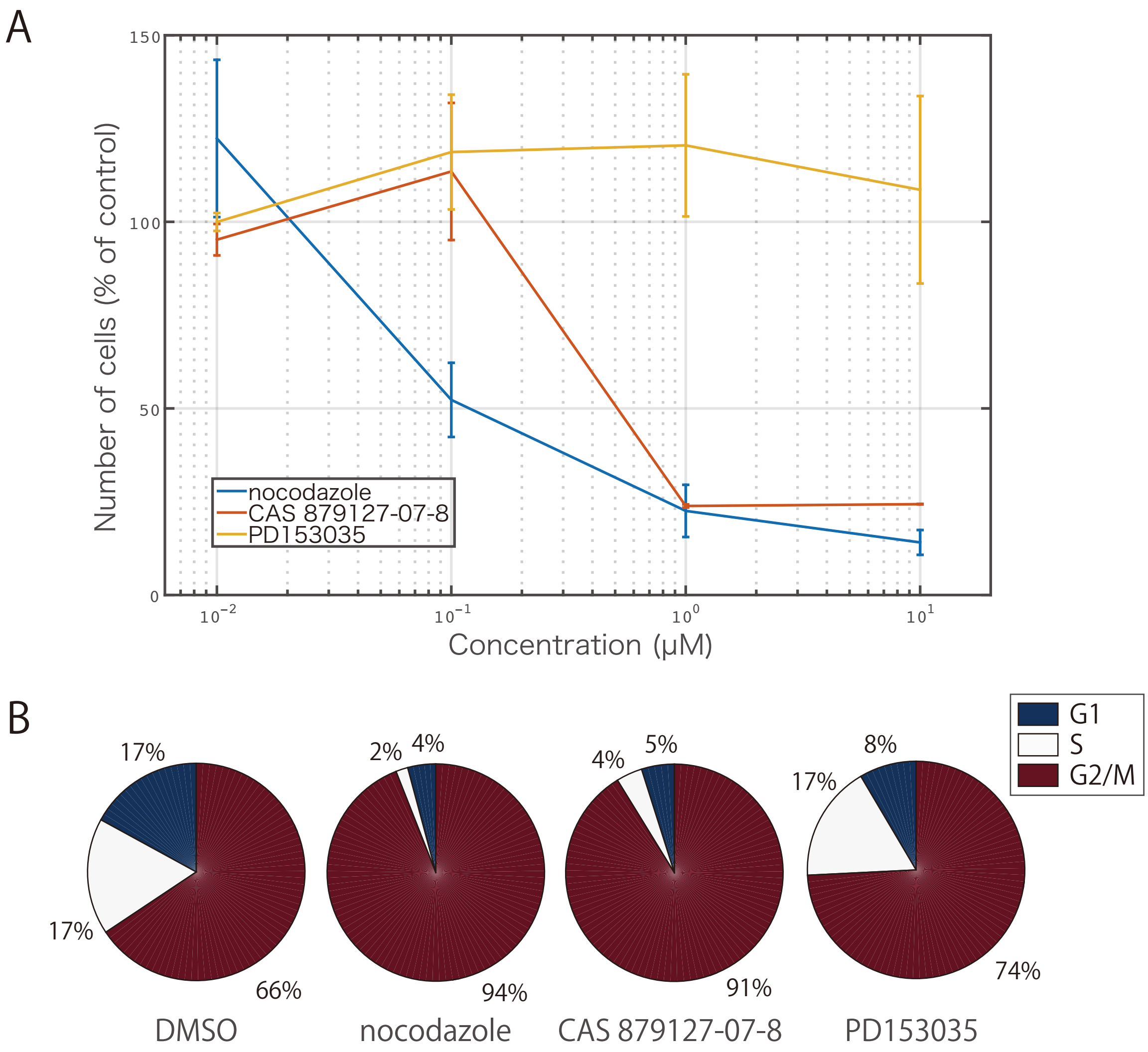

Supplementary Figure S9. Mitotic arrest was observed in CAS 879127-07-8-treated cells. Cells were treated with indicated concentration of inhibitors for 24 h, fixed, and stained with Hoechst dye. Nuclei (identified by Hoechst dye) were counted, and mean and S.D were calculated from two independent experiments (A). Each nuclei at 10µM of each compound were clasified G1, S and G2/M phase according to its signal intensities (B).

Supplementary Table S1. Descriptors used in this study. Each descriptor is the value obtained from the object measured from the indicated ROI(s). Pearson's correlation coefficients were calculated between two independent experiments.

Descriptors	Object	Measured	Regions of interest	Pearson's correlation coeffiiciencies
Cell_Size'	Cells	Area	Cells	0.535391082
'EGFR_Endosome_Size'	EGFR	Area	Cells	0.611594681
'EGF_Endosome_Size'	EGF	Area	Cells	0.477697605
'Cytoplasm_EGFR'	EGFR	Integrated intensity	Cytoplasm	0.655836959
'Perinuclear_EGFR'	EGFR	Integrated intensity	Perinuclear	0.681592243
PM_EGFR'	EGFR	Integrated intensity	PM	0.684364198
'Nucleus_EGFR'	EGFR	Integrated intensity	Nucleus	0.561892407
'Cell_EGFR'	EGFR	Integrated intensity	Cells	0.610222752
Perinuclear_to_PM_EGFR'	EGFR	Ratio of integrated intensity	Perinuclear and PM	0.63498157
PM_to_Perinuclear_EGFR'	EGFR	Ratio of integrated intensity	PM and Perinuclear	0.674129843
'Cytoplasm_pERK'	pERK	Integrated intensity	Cytoplasm	0.770445657
'Nucleus_pERK'	pERK	Integrated intensity	Nucleus	0.809894608
'Cell_pERK'	pERK	Integrated intensity	Cells	0.778845428
'Cytoplasm_EGF'	EGF	Integrated intensity	Cytoplasm	−0.002549039
'Perinuclear_EGF'	EGF	Integrated intensity	Perinuclear	0.186516278
PM_EGF'	EGF	Integrated intensity	PM	0.080172454
'Nucleus_EGF'	EGF	Integrated intensity	Nucleus	0.291248245
'Cell_EGF'	EGF	Integrated intensity	Cells	0.039132228
'Cytoplasm_EGF_EGFR_ratio'	EGF and EGFR	Ratio of integrated intensity	Cytoplasm	0.323564171
'Perinuclear_EGF_EGFR_ratio'	EGF and EGFR	Ratio of integrated intensity	Perinuclear	0.370877669
PM_EGF_EGFR_ratio'	EGF and EGFR	Ratio of integrated intensity	PM	0.253864099
'Cell_EGF_EGFR_ratio'	EGF and EGFR	Ratio of integrated intensity	Cells	0.314486024
Perinuclear_to_PM_EGF'	EGF	Ratio of integrated intensity	Perinuclear and PM	0.341756477
PM_to_Perinuclear_EGF'	EGF	Ratio of integrated intensity	PM and Perinuclear	0.416401248
'Cells_EGFR_Count'	EGFR	Count	Cells	0.690971403
'Cells_EGF_Count'	EGF	Count	Cells	0.408345981
'Cells_Ovlp_EGFR_EGF_Count'	Area overlapping EGFR and EGF	Count	Cells	0.798401787
'Cells_Ovlp_endosomes_EGFR_EGF_Count'	Endosomes containing both EGFR and EGF	Count	Cells	0.784176984
'Cytoplasm_EGFR_Count'	EGFR	Count	Cytoplasm	0.714156258
'Cytoplasm_EGF_Count'	EGF	Count	Cytoplasm	0.329887795
'Cytoplasm_EGFR_EGF_Count'	Area overlapping EGFR and EGF	Count	Cytoplasm	0.792414331
'Cytoplasm_endosomes_EGFR_EGF_Count'	Endosomes containing both EGFR and EGF	Count	Cytoplasm	0.79748211
'Perinuclear_EGFR_Count'	EGFR	Count	Perinuclear	0.732853041
'Perinuclear_EGF_Count'	EGF	Count	Perinuclear	0.540243365
'Perinuclear_EGFR_EGF_Count'	Area overlapping EGFR and EGF	Count	Perinuclear	0.737244778
'Perinuclear_endosomes_EGFR_EGF_Count'	Endosomes containing both EGFR and EGF	Count	Perinuclear	0.710919605
PM_EGFR_Count'	EGFR	Count	PM	0.87491316
PM_EGF_Count'	EGF	Count	PM	0.563985665
PM_EGFR_EGF_Count'	Area overlapping EGFR and EGF	Count	PM	0.908400996
PM_endosomes_EGFR_EGF_Count'	Endosomes containing both EGFR and EGF	Count	PM	0.915444325
'EGFR_Endosome_Size'	EGFR	Area	Cells	0.741563529
'PI3P_Size'	PI3P	Area	Cells	0.52571616
'PI4P_Size'	PI4P	Area	Cells	0.48365768
'Cytoplasm_EGFR'	EGFR	Integrated intensity	Cytoplasm	0.715403082
'Perinuclear_EGFR'	EGFR	Integrated intensity	Perinuclear	0.73127571
PM_EGFR'	EGFR	Integrated intensity	PM	0.735574401
'Nucleus_EGFR'	EGFR	Integrated intensity	Nucleus	0.61116441
'Cell_EGFR'	EGFR	Integrated intensity	Cells	0.626878059
Perinuclear_to_PM_EGFR'	EGFR	Ratio of integrated intensity	Perinuclear and PM	0.586768145
PM_to_Perinuclear_EGFR'	EGFR	Ratio of integrated intensity	PM and Perinuclear	0.74483166
'Cytoplasm_PI3P'	PI3P	Integrated intensity	Cytoplasm	0.599773934
'Perinuclear_PI3P'	PI3P	Integrated intensity	Perinuclear	0.695392794
PM_PI3P'	PI3P	Integrated intensity	PM	0.728894509
'Nucleus_PI3P'	PI3P	Integrated intensity	Nucleus	0.438772956
'Cell_PI3P'	PI3P	Integrated intensity	Cells	0.582656381
Perinuclear_to_PM_PI3P'	PI3P	Ratio of integrated intensity	Perinuclear and PM	0.552271625
PM_to_Perinuclear_PI3P'	PI3P	Ratio of integrated intensity	PM and Perinuclear	0.702111456
'Cytoplasm_PI4P'	PI4P	Integrated intensity	Cytoplasm	0.434025237
'Perinuclear_PI4P'	PI4P	Integrated intensity	Perinuclear	0.698594066
PM_PI4P'	PI4P	Integrated intensity	PM	0.286292426
'Nucleus_PI4P'	PI4P	Integrated intensity	Nucleus	0.62510275
'Cell_PI4P'	PI4P	Integrated intensity	Cells	0.527153864
Perinuclear_to_PM_PI4P'	PI4P	Ratio of integrated intensity	Perinuclear and PM	0.593527945
PM_to_Perinuclear_PI4P'	PI4P	Ratio of integrated intensity	PM and Perinuclear	0.036481085
'Cells_EGFR_Count'	EGFR	Count	Cells	0.263615588
'Cells_PI3P_Count'	PI3P	Count	Cells	0.630886912
'Cells_PI4P_Count'	PI4P	Count	Cells	0.383478789
'Cells_Ovlp_EGFR_PI3P_Count'	Area overlapping EGFR and PI3P	Count	Cells	0.290288738
'Cells_Ovlp_endosomes_EGFR_PI3P_Count'	Endosomes containing both EGFR and PI3P	Count	Cells	0.301762817
'Cells_Ovlp_EGFR_PI4P_Count'	Area overlapping EGFR and PI4P	Count	Cells	0.171489542
'Cells_Ovlp_endosomes_EGFR_PI4P_Count'	Endosomes containing both EGFR and PI4P	Count	Cells	0.156102557
'Cells_Ovlp_EGFR_PI3P_PI4P_Count'	Area overlapping EGFR, PI3P, and PI4P	Count	Cells	0.366214476
'Cells_Ovlp_endosomes_EGFR_PI3P_PI4P_Count'	Endosomes containing EGFR, PI3P, and PI4P	Count	Cells	0.318786507
'Cells_Ovlp_PI3P_PI4P_Count'	Area overlapping PI3P and PI4P	Count	Cells	0.486930074
'Cells_Ovlp_endosomes_PI3P_PI4P_Count'	Endosomes containing both PI3P and PI4P	Count	Cells	0.486564489
'Cytoplasm_EGFR_Count'	EGFR	Count	Cytoplasm	0.379121558
'Cytoplasm_PI3P_Count'	PI3P	Count	Cytoplasm	0.685087201
'Cytoplasm_PI4P_Count'	PI4P	Count	Cytoplasm	0.346539886
'Cytoplasm_Ovlp_endosomes_EGFR_PI3P_Count'	Endosomes containing both EGFR and PI3P	Count	Cytoplasm	0.234410499
'Cytoplasm_Ovlp_endosomes_EGFR_PI4P_Count'	Endosomes containing both EGFR and PI4P	Count	Cytoplasm	0.183255244
'Cytoplasm_Ovlp_endosomes_EGFR_PI3P_PI4P_Count'	Endosomes containing EGFR, PI3P, and PI4P	Count	Cytoplasm	0.324745348
'Cytoplasm_Ovlp_endosomes_PI3P_PI4P_Count'	Endosomes containing both PI3P and PI4P	Count	Cytoplasm	0.431861309
'Perinuclear_EGFR_Count'	EGFR	Count	Perinuclear	0.280356508
'Perinuclear_PI3P_Count'	PI3P	Count	Perinuclear	0.67613279
'Perinuclear_PI4P_Count'	PI4P	Count	Perinuclear	0.455288277
'Perinuclear_Ovlp_endosomes_EGFR_PI3P_Count'	Endosomes containing both EGFR and PI3P	Count	Perinuclear	0.295785008
'Perinuclear_Ovlp_endosomes_EGFR_PI4P_Count'	Endosomes containing both EGFR and PI4P	Count	Perinuclear	0.277069677
'Perinuclear_Ovlp_endosomes_EGFR_PI3P_PI4P_Count'	Endosomes containing EGFR, PI3P, and PI4P	Count	Perinuclear	0.295503152
'Perinuclear_Ovlp_endosomes_PI3P_PI4P_Count'	Endosomes containing both PI3P and PI4P	Count	Perinuclear	0.26186344
PM_EGFR_Count'	EGFR	Count	PM	0.702596288
PM_PI3P_Count'	PI3P	Count	PM	0.719842708
PM_PI4P_Count'	PI4P	Count	PM	−0.011834707
PM_Ovlp_endosomes_EGFR_PI3P_Count'	Endosomes containing both EGFR and PI3P	Count	PM	0.28613878
PM_Ovlp_endosomes_EGFR_PI4P_Count'	Endosomes containing both EGFR and PI4P	Count	PM	0.22175166
PM_Ovlp_endosomes_EGFR_PI3P_PI4P_Count'	Endosomes containing EGFR, PI3P, and PI4P	Count	PM	0.226707428
PM_Ovlp_endosomes_PI3P_PI4P_Count'	Endosomes containing both PI3P and PI4P	Count	PM	0.187167888
Cell_Size'	Cells	Area	Cells	0.813280728
'EGFR_Endosome_Size'	EGFR	Area	Cells	0.52007986
'PIP2_Size'	PIP2	Area	Cells	0.202643892
'Cytoplasm_EGFR'	EGFR	Integrated intensity	Cytoplasm	0.727948497
'Perinuclear_EGFR'	EGFR	Integrated intensity	Perinuclear	0.806565377
PM_EGFR'	EGFR	Integrated intensity	PM	0.703062952
'Nucleus_EGFR'	EGFR	Integrated intensity	Nucleus	0.651085187

'Cell_EGFR'	EGFR	Integrated intensity	Cells	0.738418377
Perinuclear_to_PM_EGFR'	EGFR	Ratio of integrated intensity	Perinuclear and PM	0.78672988
PM_to_Perinuclear_EGFR'	EGFR	Ratio of integrated intensity	PM and Perinuclear	0.800252357
'Cytoplasm_PIP2'	PIP2	Integrated intensity	Cytoplasm	0.320198398
'Perinuclear_PIP2'	PIP2	Integrated intensity	Perinuclear	0.507619101
PM_PIP2'	PIP2	Integrated intensity	PM	0.440713748
'Nucleus_PIP2'	PIP2	Integrated intensity	Nucleus	0.633727919
'Cell_PIP2'	PIP2	Integrated intensity	Cells	0.387844405
Cell_Size'	Cells	Area	Cells	0.648216122
'EGFR_Endosome_Size'	EGFR	Area	Cells	0.69810245
'Tfn_Endosome_Size'	Tfn	Area	Cells	0.651946023
'Cytoplasm_EGFR'	EGFR	Integrated intensity	Cytoplasm	0.606123966
'Perinuclear_EGFR'	EGFR	Integrated intensity	Perinuclear	0.735428135
PM_EGFR'	EGFR	Integrated intensity	PM	0.743938373
'Nucleus_EGFR'	EGFR	Integrated intensity	Nucleus	0.664656512
'Cell_EGFR'	EGFR	Integrated intensity	Cells	0.60461786
Perinuclear_to_PM_EGFR'	EGFR	Ratio of integrated intensity	Perinuclear and PM	0.703527242
PM_to_Perinuclear_EGFR'	EGFR	Ratio of integrated intensity	PM and Perinuclear	0.742535314
'Cytoplasm_pAkt'	pAkt	Integrated intensity	Cytoplasm	0.914316144
'Perinuclear_pAkt'	pAkt	Integrated intensity	Perinuclear	0.92971399
PM_pAkt'	pAkt	Integrated intensity	PM	0.940217119
'Nucleus_pAkt'	pAkt	Integrated intensity	Nucleus	0.908207105
'Cell_pAkt'	pAkt	Integrated intensity	Cells	0.921508053
PM_to_Perinuclear_pAkt'	pAkt	Ratio of integrated intensity	PM and Perinuclear	0.925924615
'Cytoplasm_Tfn'	Tfn	Integrated intensity	Cytoplasm	0.347340291
'Perinuclear_Tfn'	Tfn	Integrated intensity	Perinuclear	0.525356535
PM_Tfn'	Tfn	Integrated intensity	PM	0.588875997
'Nucleus_Tfn'	Tfn	Integrated intensity	Nucleus	0.599167547
'Cell_Tfn'	Tfn	Integrated intensity	Cells	0.381330645
Perinuclear_to_PM_Tfn'	Tfn	Ratio of integrated intensity	Perinuclear and PM	0.49231075
PM_to_Perinuclear_Tfn'	Tfn	Ratio of integrated intensity	PM and Perinuclear	0.654934139
'Cells_EGFR_Count'	EGFR	Count	Cells	0.651060385
'Cells_Transferrin_Count'	Tfn	Count	Cells	0.599081651
'Cells_Ovlp_EGFR_Tfn_Count'	Area overlapping EGFR and Tfn	Count	Cells	0.789554396
'Cells_Ovlp_endosomes_EGFR_Tfn_Count'	Endosomes containing both EGFR and Tfn	Count	Cells	0.790422243
'Cytoplasm_EGFR_Count'	EGFR	Count	Cytoplasm	0.684322216
'Cytoplasm_Transferrin_Count'	Tfn	Count	Cytoplasm	0.619266918
'Cytoplasm_EGFR_Tfn_Count'	Area overlapping EGFR and Tfn	Count	Cytoplasm	0.780417253
'Cytoplasm_endosomes_EGFR_Tfn_Count'	Endosomes containing both EGFR and Tfn	Count	Cytoplasm	0.787224764
'Perinuclear_EGFR_Count'	EGFR	Count	Perinuclear	0.784409191
'Perinuclear_Transferrin_Count'	Tfn	Count	Perinuclear	0.553305737
'Perinuclear_EGFR_Tfn_Count'	Area overlapping EGFR and Tfn	Count	Perinuclear	0.699792425
'Perinuclear_endosomes_EGFR_Tfn_Count'	Endosomes containing both EGFR and Tfn	Count	Perinuclear	0.713114744
PM_EGFR_Count'	EGFR	Count	PM	0.817698775
PM_Transferrin_Count'	Tfn	Count	PM	0.750236742
PM_EGFR_Tfn_Count'	Area overlapping EGFR and Tfn	Count	PM	0.878140445
PM_endosomes_EGFR_Tfn_Count'	Endosomes containing both EGFR and Tfn	Count	PM	0.858208289

Supplementary Table S2. Descriptors selected on the basis of Pearson's correlation coefficient (R > 0.4). These descriptors were used for subsequent principal component analysis.

Selected Descriptors

'Cell_Area'
'EGFR_Endosome_Size'
'EGF_Endosome_Size'
'Cytoplasm_EGFR'
'Perinuclear_EGFR'
'Peripheral_EGFR'
'Nucleus_EGFR'
'Cell_EGFR'
'Perinuclear_to_Peripheral_EGFR'
'Peripheral_to_Perinuclear_EGFR'
'Cytoplasm_pERK'
'Nucleus_pERK'
'Cell_pERK'
'Cytoplasm_EGF_EGFR_ratio'
'Perinuclear_EGF_EGFR_ratio'
'Cell_EGF_EGFR_ratio'
'Peripheral_to_Perinuclear_EGF'
'Cells_EGFR_Count'
'Cells_EGF_Count'
'Cells_Ovlp_EGFR_EGF_Count'
'Cells_Ovlp_endosomes_EGFR_EGF_Count'
'Cytoplasm_EGFR_Count'
'Cytoplasm_EGFR_EGF_Count'
'Cytoplasm_endosomes_EGFR_EGF_Count'
'Perinuclear_EGFR_Count'
'Perinuclear_EGF_Count'
'Perinuclear_EGFR_EGF_Count'
'Perinuclear_endosomes_EGFR_EGF_Count'
'Peripheral_EGFR_Count'
'Peripheral_EGF_Count'
'Peripheral_EGFR_EGF_Count'
'Peripheral_endosomes_EGFR_EGF_Count'
'Cell_number'
'EGFR_Endosome_Size'
'PI3P_Size'
'PI4P_Size'
'Cytoplasm_EGFR'
'Perinuclear_EGFR'
'Peripheral_EGFR'
'Nucleus_EGFR'
'Cell_EGFR'
'Perinuclear_to_Peripheral_EGFR'
'Peripheral_to_Perinuclear_EGFR'
'Cytoplasm_PI3P'
'Perinuclear_PI3P'
'Peripheral_PI3P'
'Nucleus_PI3P'
'Cell_PI3P'
'Perinuclear_to_Peripheral_PI3P'
'Peripheral_to_Perinuclear_PI3P'
'Cytoplasm_PI4P'
'Perinuclear_PI4P'
'Nucleus_PI4P'
'Cell_PI4P'
'Perinuclear_to_Peripheral_PI4P'
'Cells_PI3P_Count'
'Cells_Ovlp_PI3P_PI4P_Count'
'Cells_Ovlp_endosomes_PI3P_PI4P_Count'
'Cytoplasm_EGFR_Count'
'Cytoplasm_PI3P_Count'
'Cytoplasm_Ovlp_endosomes_PI3P_PI4P_Count'
'Perinuclear_PI3P_Count'
'Perinuclear_PI4P_Count'
'Peripheral_EGFR_Count'
'Peripheral_PI3P_Count'
'Cell_Area'
'EGFR_Endosome_Size'
'Cytoplasm_EGFR'
'Perinuclear_EGFR'
'Peripheral_EGFR'
'Nucleus_EGFR'
'Cell_EGFR'
'Perinuclear_to_Peripheral_EGFR'
'Peripheral_to_Perinuclear_EGFR'
'Perinuclear_PIP2'
'Peripheral_PIP2'
'Nucleus_PIP2'

'Cell_Area'
'EGFR_Endosome_Size'
'Tfn_Endosome_Size'
'Cytoplasm_EGFR'
'Perinuclear_EGFR'
'Peripheral_EGFR'
'Nucleus_EGFR'
'Cell_EGFR'
'Perinuclear_to_Peripheral_EGFR'
'Peripheral_to_Perinuclear_EGFR'
'Cytoplasm_pAkt'
'Perinuclear_pAkt'
'Peripheral_pAkt'
'Nucleus_pAkt'
'Cell_pAkt'
'Peripheral_to_Perinuclear_pAkt'
'Perinuclear_Tfn'
'Peripheral_Tfn'
'Nucleus_Tfn'
'Perinuclear_to_Peripheral_Tfn'
'Peripheral_to_Perinuclear_Tfn'
'Cells_EGFR_Count'
'Cells_Transferrin_Count'
'Cells_Ovlp_EGFR_Tfn_Count'
'Cells_Ovlp_endosomes_EGFR_Tfn_Count'
'Cytoplasm_EGFR_Count'
'Cytoplasm_Transferrin_Count'
'Cytoplasm_EGFR_Tfn_Count'
'Cytoplasm_endosomes_EGFR_Tfn_Count'
'Perinuclear_EGFR_Count'
'Perinuclear_Transferrin_Count'
'Perinuclear_EGFR_Tfn_Count'
'Perinuclear_endosomes_EGFR_Tfn_Count'
'Peripheral_EGFR_Count'
'Peripheral_Transferrin_Count'
'Peripheral_EGFR_Tfn_Count'
'Peripheral_endosomes_EGFR_Tfn_Count'
